# Vangl2 deficient zebrafish exhibit hallmarks of neural tube closure defects

**DOI:** 10.1101/2023.11.09.566412

**Published:** 2023-11-09

**Authors:** Jacalyn MacGowan, Mara Cardenas, Margot Kossmann Williams

**Affiliations:** 1 Center for Precision Environmental Health, Baylor College of Medicine, Houston, TX; 2 Department of Molecular and Cellular Biology, Baylor College of Medicine, Houston, TX; 3 Center for Cell and Gene Therapy, Baylor College of Medicine, Houston, TX; 4 Stem Cells and Regenerative Medicine Center, Baylor College of Medicine, Houston, TX

**Keywords:** Neurulation, Neural tube defects, Planar cell polarity, Zebrafish

## Abstract

Neural tube defects (NTDs) are among the most devastating and common congenital anomalies worldwide, and the ability to model these conditions *in vivo* is essential for identifying causative genetic and environmental factors. Although zebrafish are ideal for rapid candidate testing, their neural tubes develop primarily via a solid neural keel rather that the fold-and-fuse method employed by mammals, raising questions about their suitability as an NTD model. Here, we demonstrate that despite outward differences, zebrafish anterior neurulation closely resembles that of mammals. For the first time, we directly observe fusion of the bilateral neural folds to enclose a lumen in zebrafish embryos. The neural folds fuse by zippering between multiple distinct but contiguous closure sites. Embryos lacking *vangl2*, a core planar cell polarity and NTD risk gene, exhibit delayed neural fold fusion and abnormal neural groove formation, yielding distinct openings and midline bifurcations in the developing neural tube. These data provide direct evidence for fold-and-fuse neurulation in zebrafish and its disruption upon loss of an NTD risk gene, highlighting conservation of vertebrate neurulation and the utility of zebrafish for modeling NTDs.

## Introduction

Neural tube defects (NTDs) such as spina bifida and anencephaly are among the most common and devastating congenital anomalies, affecting approximately 1 in 1,000 births in the United States (1) and even more worldwide (2). These conditions result from incomplete closure of the neural tube during embryogenesis, often leaving the neural tube lumen open to the outside of the body. While folate supplementation has greatly reduced the incidence of NTDs, as many as 70% of cases are folate-resistant (3). Mutations in a number of genes are associated with an increased likelihood of NTDs (4), but are not the sole risk factors for NTD (5, 6). Additional potential risk factors include mutations in multiple risk genes (7), certain teratogenic drugs (8, 9), and exposure to environmental toxicants (10-16), implicating complex gene-environment interactions in their etiology. Indeed, the stalled decline in NTDs after folate fortification in the U.S. (3) demonstrates that additional risk factors remain to be discovered. One major limitation is the inability to draw causal relationships between reported risk factors and NTD occurrence. Some genetic and/or environmental risk factors identified from patient cohorts have been shown to cause NTDs in mouse models (17-19). However, the small litter sizes, high husbandry costs, and large space requirements of mice are prohibitive of large-scale genetic and/or chemical screening that could convert associations into causal relationships, highlighting the need for additional animal models of NTDs.

Due to their relatively low husbandry costs, rapid external development, large clutch sizes, and amenability to genetic manipulation, zebrafish are an ideal vertebrate model for genetic and chemical screens to identify causes of NTDs. However, the morphology of the developing neural tube differs substantially between zebrafish and amniote species. Primary neurulation in chick, mouse, and human embryos is driven by convergent extension (CE) of the developing neural plate followed by formation of hinge points (20-27) that elevate the bilateral neural folds and bend them toward each other (26, 28, 29). The neural folds then meet at the dorsal midline and fuse by zippering between discrete closure points (30, 31), completing the “fold-and-fuse” process that encloses the neural tube lumen. By contrast, the zebrafish spinal cord develops from the neural keel, a solid structure that later undergoes cavitation to form a central lumen (32, 33). The site of this lumen is established through a series of midline-crossing mitoses termed “C-divisions” which distribute one daughter cell of each side of the neural keel midline (32, 34-39). For this reason, primary neurulation in zebrafish has been likened to secondary neurulation in amniote embryos (33, 40, 41). Zebrafish were therefore viewed not only as questionable for NTD modeling, but also as fundamentally different from other vertebrates in their mechanism of neurulation.

However, more recent findings reveal that despite these outward differences, several hallmarks of primary neurulation are conserved in zebrafish. For example, CE morphogenesis narrows the neural plate (38, 42), and apical constriction at the midline forms a medial hinge point-like structure (43, 44). The neural folds were also shown to zipper closed in the forebrain region of zebrafish (44) in a fashion strikingly similar to mice (30, 31). These conserved neurulation mechanisms open the possibility of modeling NTDs, or aspects thereof, in the experimentally tractable zebrafish model. Indeed, previous studies have proposed bifurcation of pineal gland precursors and/or the dorsal roof plate as proxies for NTDs in zebrafish (45-48). These phenotypes are suggestive of reduced neural fold convergence, but it is unclear whether they exhibit other hallmarks of NTDs, such as lumens that remain open to the outside. Furthermore, bifurcated pineal and roof plate domains resulted from reduced Nodal signaling and N-cadherin function (45-48), but mutations in Nodal signaling components are associated with holoprosencephaly in human patients (49-51) rather than NTDs. Whether these or other NTD-like phenotypes are induced by loss of human NTD risk genes in zebrafish was unknown.

Among mutations known or suspected to cause NTDs in mice and humans, respectively, many affect components of planar cell polarity (PCP) signaling, a highly conserved regulator of vertebrate morphogenesis. Loss of PCP genes like *Vangl2* disrupts both CE of the neural plate and hinge point formation, preventing anteroposterior axis elongation and neural tube closure in mouse, chick, and *Xenopus* (20, 21, 52-65). Mutations in PCP genes (including *VANGL2*) are also associated with NTDs in several patient cohorts (7, 66-71). Loss of PCP signaling similarly disrupts CE and neural tube development in zebrafish (38, 42, 72-81), but these phenotypes differ from amniote NTDs due to differences in neurulation. For example, C-divisions (which determine the site of lumen formation) are disorganized In PCP mutant *vangl2* / *trilobite (tri)−/−* zebrafish, giving rise to ectopic neuroectodermal structures and neural tube lumens (38, 39, 72). Because these phenotypes do not outwardly resemble NTDs in other vertebrate species or humans (i.e. no lumens that remain open to the outside), it was broadly assumed that zebrafish neurulate via fundamentally different mechanisms than other vertebrates and are poor models for NTD research.

Here, we reevaluate the neural tube phenotypes of *vangl2* deficient zebrafish embryos with a focus on the brain region at early stages of neurulation. By examining neural tube development at the time of neural fold zippering, we found that many *vangl2*-deficient embryos exhibit a Distinct Opening of the Neural Tube (DONuT), a 3-dimensional pit-shaped structure in the forebrain region. DONuT formation correlates with the severity of axis extension defects and delayed midline convergence of pineal precursors, linking it to reduced CE morphogenesis. Live time-lapse imaging of neural tube closure revealed that, in addition to the previously described zippering of the anterior neural folds, wild-type (WT) zebrafish exhibit a distinct but contiguous posterior site of neural fold fusion. These two sites zipper in opposite directions from a central point of contact to close the anterior neural tube. Using optical transverse sectioning of live embryos, we further showed that the bilateral neural folds fuse to enclose a lumen in a process strikingly similar to amniote neurulation. Moreover, neural fold fusion is delayed in *vangl2* deficient embryos, reflecting reduced CE cell movements and abnormal neural groove formation. Together, these data provide direct evidence for fold-and-fuse neurulation in zebrafish and show that this process requires Vangl2. This demonstrates the deep conservation of neurulation mechanisms among vertebrates and highlights the potential utility of zebrafish for modeling the morphogenetic processes underlying neural tube closure and NTDs.

## Results

### Bifurcated pineal precursors and roof plates reflect abnormal anterior neural tube morphology in Vangl2 deficient zebrafish embryos.

**Figure 1.**
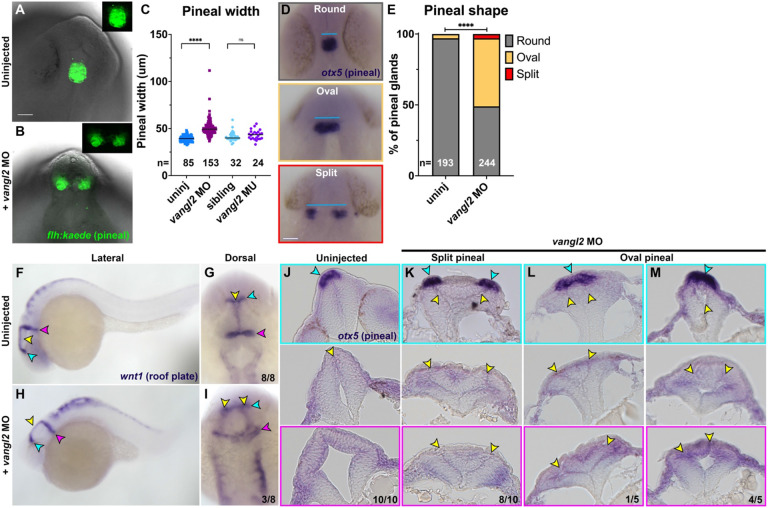
Loss of *vangl2* disrupts pineal and neural roof plate morphology. A-B) Example images of pineal precursor morphology in Tg[*flh:kaede*] control (A) or *vangl2* MO-injected (B) embryos at 28 hpf, viewed from the dorsal side. C) Width of pineal precursor domains in embryos of the conditions indicated, as measured from *otx5* whole mount in situ hybridization (WISH) at 28 hpf (D, blue lines). Each dot represents a single embryo, black bars are median values. ****p<0.0001, ns p=0.10, Mann-Whitney tests. E) Classification of pineal shape in 28 hpf control and *vangl2* MO-injected embryos expressing *flh:kaede* or WISH stained for *otx5*. n values indicate the number of embryos of each condition measured from at least 3 independent trials. ****p<0.0001, Chi-square test. Scale bars — 50 μm. F-I) WISH for roof plate marker *wnt1* at 28 hpf in control (F-G) and *vangl2* MO-injected (H-I) embryos. Yellow arrowheads indicate the midline roof plate, cyan arrowheads indicate the epithalamus, and magenta arrowheads indicate the mid-hindbrain boundary (MHB). J-M) Transverse histological sections through the anterior neural tube at the level of the epithalamus (top panels), midbrain (middle panels), and MHB (bottom panels) in 28 hpf embryos of the conditions indicated. Cyan arrowheads indicate pineal precursors stained by *otx5* WISH. Yellow arrowheads indicate the neural tube midline(s)/lumen(s). Fractions indicate the number of embryos with the depicted phenotype over the total number of embryos examined for each condition. Anterior is up in (A,B,D,G,I), dorsal is up in (F,H,J-M).

Bifurcation of pineal gland precursors and the dorsal roof plate around 24 hours post fertilization (hpf) were previously suggested as proxies for NTDs in zebrafish embryos with disrupted Nodal signaling or mutations in *cdh2* (encoding N-cadherin) (45-48). To determine whether such phenotypes were present in zebrafish embryos lacking the PCP signaling component (and ortholog of a human NTD risk gene (70, 71)) *vangl2*, we examined the morphology of these structures in *vangl2/trilobite (tri*) homozygous mutants, *vangl2* morphants, and sibling controls at approximately 28 hpf. Using both a transgenic *flh:kaede* (82) line in which pineal precursors fluoresce green (Fig. 1A-B) and whole mount *in situ* hybridization (WISH) for the pineal marker *otx5* (Fig. 1D), we found that embryos injected with 2 ng *vangl2* morpholino oligonucleotide (MO) (83) exhibited significantly wider pineal domains than control siblings when measured from the lateral-most edges (Fig. 1C) and were significantly more likely to have split or elongated oval-shaped pineal domains than controls (Fig. 1E). Notably, homozygous *vangl2/trilobite (tri*)−/− embryos did not exhibit increased pineal width at this stage (Fig. 1C). This is likely due to maternally deposited *vangl2* that is eliminated by the MO (42, 84), consistent with more severe phenotypes in maternal zygotic (MZ)*vangl2*−/− than zygotic (Z)*vangl2*−/− embryos (72). WISH for the dorsal neural tube marker *wnt1* also revealed bifurcated roof plates in a subset *vangl2* morphants (Fig. 1F-I). Histological analysis of 28 hpf *vangl2* morphant anterior neural tubes (fore- through hind-brain) with split or oval-shaped pineal domains revealed ectopic tissue and supernumerary midline structures (Fig. 1J-M, yellow arrows) similar to those reported in the spinal cord region of *vangl2* deficient embryos (38, 39, 72). Eight of the ten embryos with split pineal domains contained ectopic midline tissue at all rostrocaudal positions examined, including at the level of the pineal (Fig. 1K). Among the embryos examined with oval-shaped pineals, only one contained ectopic midline tissue at the level of the pineal (Fig. 1L) while the remaining four contained a single midline in the rostral-most sections but ectopic midlines further rostrally (Fig. 1M). This suggests that split pineal domains are associated with ectopic midline tissues resulting from abnormal C-divisions, but that the anterior neural tube is less sensitive to loss of *vangl2* than more posterior regions.

We compared these phenotypes to those of Nodal signaling-deficient embryos, in which split pineal domains were hypothesized to represent open neural tubes (45-47). As previously reported, embryos completely lacking Nodal signaling through maternal and zygotic loss of the Nodal coreceptor *oep/tdgf1* (MZ*oep*−/−) or treated with the Nodal inhibitor SB505124 at either sphere or dome stage (4-4.3 hpf) exhibited substantially wider and split pineal domains (Supp. Fig. 1A-E). Later SB505124 treatment at 30% epiboly (4.7 hpf) yielded embryos with a range of pineal phenotypes from split to round, as seen previously (46). Histological analysis of SB505124-treated embryos at 28 hpf revealed a striking Swiss cheese-like pattern of multiple small holes in the neural tube of every embryo examined, regardless of whether their pineal domains were split or closed (Supp. Fig. 1F-H). This internal anatomy of the neural tube is consistent with previous reports of multiple lumen-like structures in MZ*oep*−/− embryos (85), and is distinct from the ectopic bilateral midlines observed in *vangl2* morphants (Fig.1J-M) despite similar pineal phenotypes. This demonstrates that bifurcation of the pineal precursors and roof plate externally can be underlain by multiple distinct internal phenotypes, which do not appear to share features with amniote NTDs.

### Vangl2 deficient embryos exhibit delayed pineal precursor convergence and distinct openings of the neural tube.

**Figure 2.**
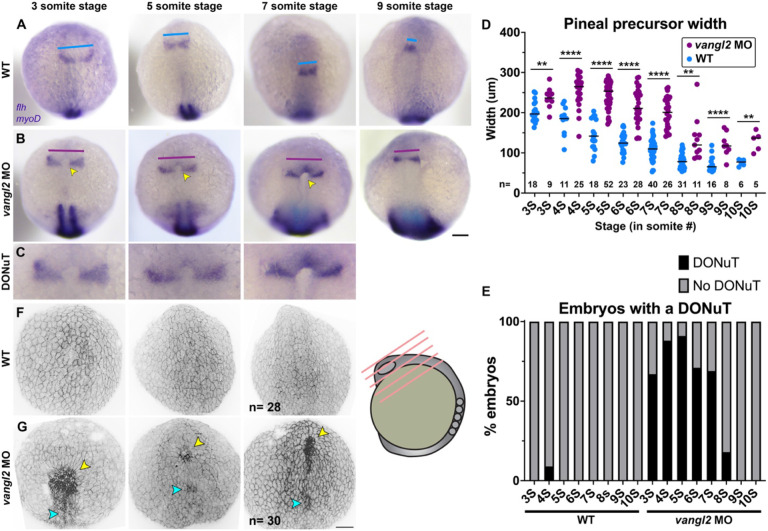
*vangl2* deficient embryos exhibit delayed pineal convergence and a Distinct Opening of the Neural Tube (DONuT). **A-B**) Representative images of pineal precursors (*flh* WISH) and somites/adaxial cells (*myoD* WISH) in control (A) and *vangl2* MO-injected (B) embryos at the stages indicated, viewed dorsally. Blue and burgundy lines indicate pineal precursor width, yellow arrowheads indicate the presence of a DONuT. **C**) Enlargements of the DONuTs from images in (B). **D**) Width of pineal precursor domains (as shown in A-B) in control (blue) and *vangl2* morphant (burgundy) embryos at the stages indicated. Each dot represents a single embryo, black bars are median values. n values indicate the number of embryos of each stage/condition measured from 3 independent trials, **p=0.003, p=0.008, p=0.007, ****p<0.0001, T-test. **E**) Percentage of embryos (as shown in A-B) of the stage/condition indicated with (black) or without (gray) a DONuT. **F-G**) 3-dimensional reconstructions of confocal Z-stacks through the anterior neural plate of fixed and phalloidin-stained embryos of the stages and conditions indicated. Yellow arrowheads indicate a DONuT, cyan arrowheads indicate openings in more posterior regions. n values indicate the number of embryos examined for each condition from 3 independent trials. Anterior is up in all images, scale bars = 100 μm.

Previous analysis of the spinal cord region in *vangl2* deficient embryos determined that ectopic tissue at the neural tube midline results from abnormal C-divisions (38, 39, 72) that normally distribute pairs of neural progenitor cells on either side of the neural midline (32, 34, 37) but occur ectopically in the absence of *vangl2*. We frequently observed a tissue mass between the split pineal domains of *vangl2* morphants (Fig. 1K), raising the possibility that ectopic midline tissue prevents pineal fusion. However, WT pineal fusion and anterior neural tube closure (at the 7-somite stage (44, 45)) precede C-divisions (at approximately 10-somite stage (34, 35, 41)), and are therefore unlikely to be caused by abnormal cell divisions. To identify the developmental origins of pineal phenotypes in embryos lacking *vangl2*, we examined neural development at the time of neural fold zippering (44) from 3-10 somite stages. In control embryos, pineal precursors marked by *flh/noto* expression began as bilateral domains that converged at the midline and fused at approximately 7 somite stage (Fig. 2A, D), as previously described (45). In *vangl2* morphant (and to a lesser degree, *tri*−/− mutant) embryos, we found that this convergence was delayed, as reflected by the increased width of *flh*+ domains across several time points (Fig. 2B, D; Supp. Fig. 2A).

However, we observed an even more striking phenotype in *vangl2* morphant embryos during this analysis: a distinct, horseshoe-shaped pit in the neural plate between the *flh*+ pineal domains (Fig. 2B-C). This structure was present in most *vangl2* morphant embryos from 3-6 somite stages, and although its incidence decreased as development proceeded, it persisted in approximately 20% of *vangl2* morphants at the 8-somite stage (Fig. 2D). This pit-like structure was not common to all embryos whose pineal precursors had not yet fused, as only one control embryo (of 163 examined) possessed such a structure even at pre-pineal fusion stages. Although the pineal domains of *vangl2/ tri−/−* embryos were wider than their WT and heterozygous controls, none exhibited a pit in their forebrain region (Supp. Fig. 2A-B), consistent with more severe phenotypes in *vangl2* morphants than mutants. 3-dimensional reconstructions of these structures from confocal Z-stacks of fixed *vangl2* morphant and control embryos stained with phalloidin (Fig. 2F-G) corroborated our WISH images. At 3-5 somite stages, this structure resembled a pit that was open to the exterior of the embryo (Fig. 2G, yellow arrowheads). Many *vangl2* morphants also possessed a second, more posteriorly positioned pit structure (Fig. 2G, cyan arrowheads). By contrast, the anterior neural plate of control embryos was covered by periderm (which develops from and is sometimes referred to as the enveloping layer (EVL)) and appeared smooth (Fig. 2F). This smooth appearance persisted to the 7-somite stage, whereas *vangl2* morphant embryos exhibited openings from which rounded cells protruded to the embryo’s exterior. Because these finding provide direct evidence for open neural tubes in *vangl2* morphant embryos, we termed this pit-shaped structure the Distinct Opening of the Neural Tube (DONuT).

### Anterior neural tube openings correlate with severity of convergent extension defects.

**Figure 3.**
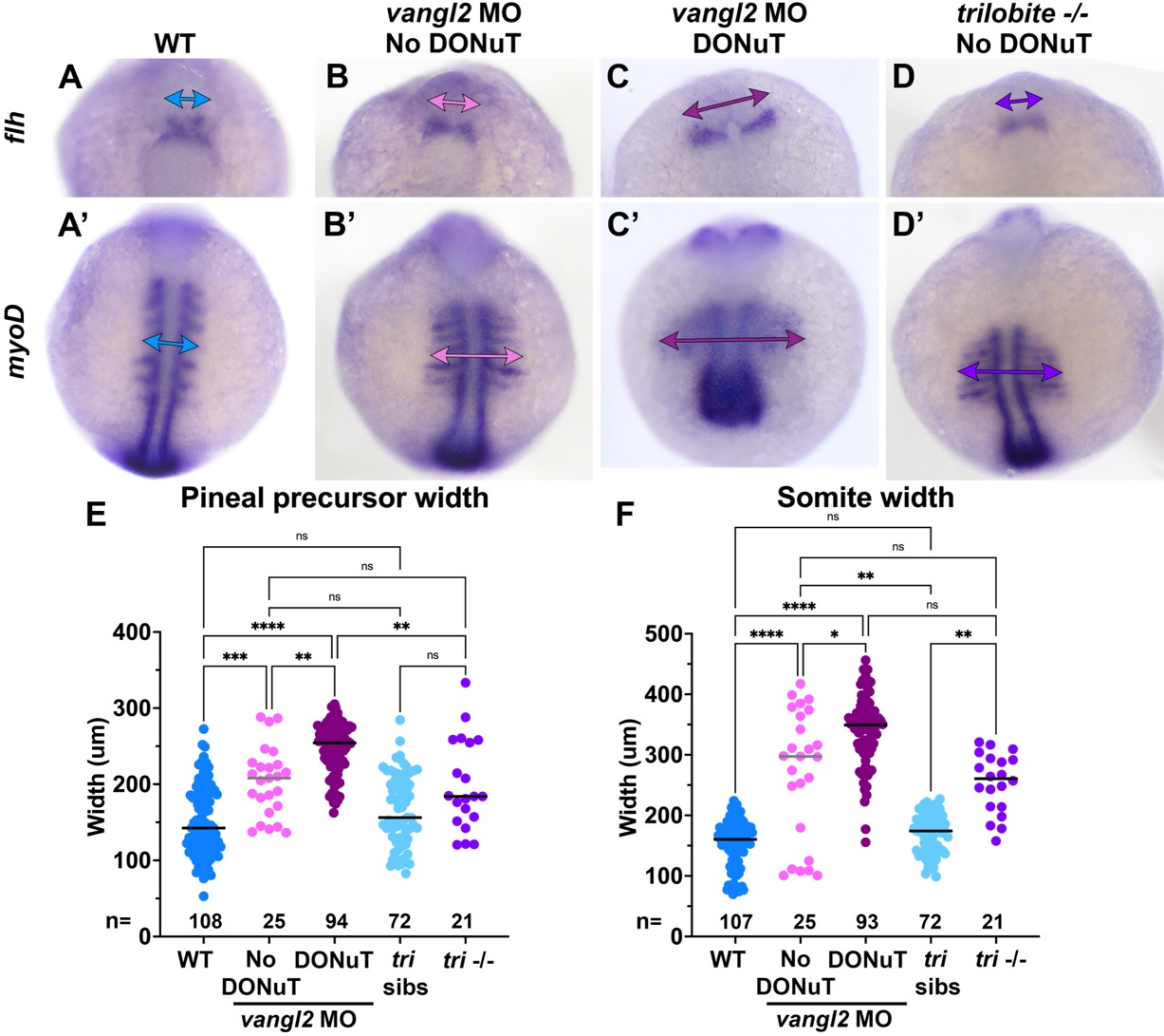
DONuT formation is correlated with severity of convergent extension defects. **A-D’**) Representative images of pineal precursors (*flh* WISH) and somites/adaxial cells (*myoD* WISH) in the same control (A), *vangl2* MO-injected (B-C), and *vangl2/ tri*−/− (D) embryos at the 7-somite stage, viewed dorsally. Double arrows indicate pineal precursor width (A-D) or somite width (A’-D’). **E-F**) Width of pineal precursor domains (E, as shown in A-D) and somites (F, as shown in A'-D') in control (blue), *tri−/−* (purple), and *vangl2* morphant embryos with (burgundy) or without (pink) a DONuT at 3-7 somite stages. Each dot represents a single embryo, black/gray bars are median values. ****p<0.0001, ***p<0.001, **p<0.01, *p<0.05, Kruskal-Wallis test with multiple comparisons. n values indicate the number of embryos of each stage/condition measured from 3 independent trials. Anterior is up in all images.

*Vangl2* deficient embryos have well described defects in CE morphogenesis during gastrulation (42, 78, 84), and their neural tube phenotypes at later stages are thought to be secondary to reduced CE (38). We hypothesized that defective CE also underlies the observed delay in convergence of the bilateral pineal precursors, which in-turn manifests as a DONuT. Consistent with this hypothesis, we found that both the *flh*+ pineal domains and *myoD*+ somites were significantly wider (consistent with reduced CE) in 3-7 somite staged *vangl2* morphants with a DONuT than those without a DONuT (Fig. 3A-C, E-F). Notably, the width of pineal domains and somites of *vangl2/ tri−/−* embryos did not differ from morphants without DONuTs but were narrower than morphants with DONuTs (Fig. 3D, E-F). This is consistent with our failure to observe DONuTs in mutant embryos, and with previous findings that *Zvangl2*−/− CE phenotypes are less severe than those of MZ*vangl2*−/− and *vangl2* morphants (72). Together, these findings indicate that embryos with more severe CE defects are more likely to exhibit openings in the neural tube, strongly implicating reduced CE during gastrulation in neural tube closure. We further speculate that some *vangl2* deficient embryos can never overcome this delay to successfully fuse the anterior neural folds, and that any embryos with a persistent DONuT and/or whose pineal domains have not yet fused by the onset of C-divisions around 10 somite stage go on to become the minority of embryos with split pineal precursors and/or roof plates at 28 hpf (Fig. 1).

### Zebrafish neural folds fuse by zippering of distinct anterior and posterior openings.

**Figure 4.**
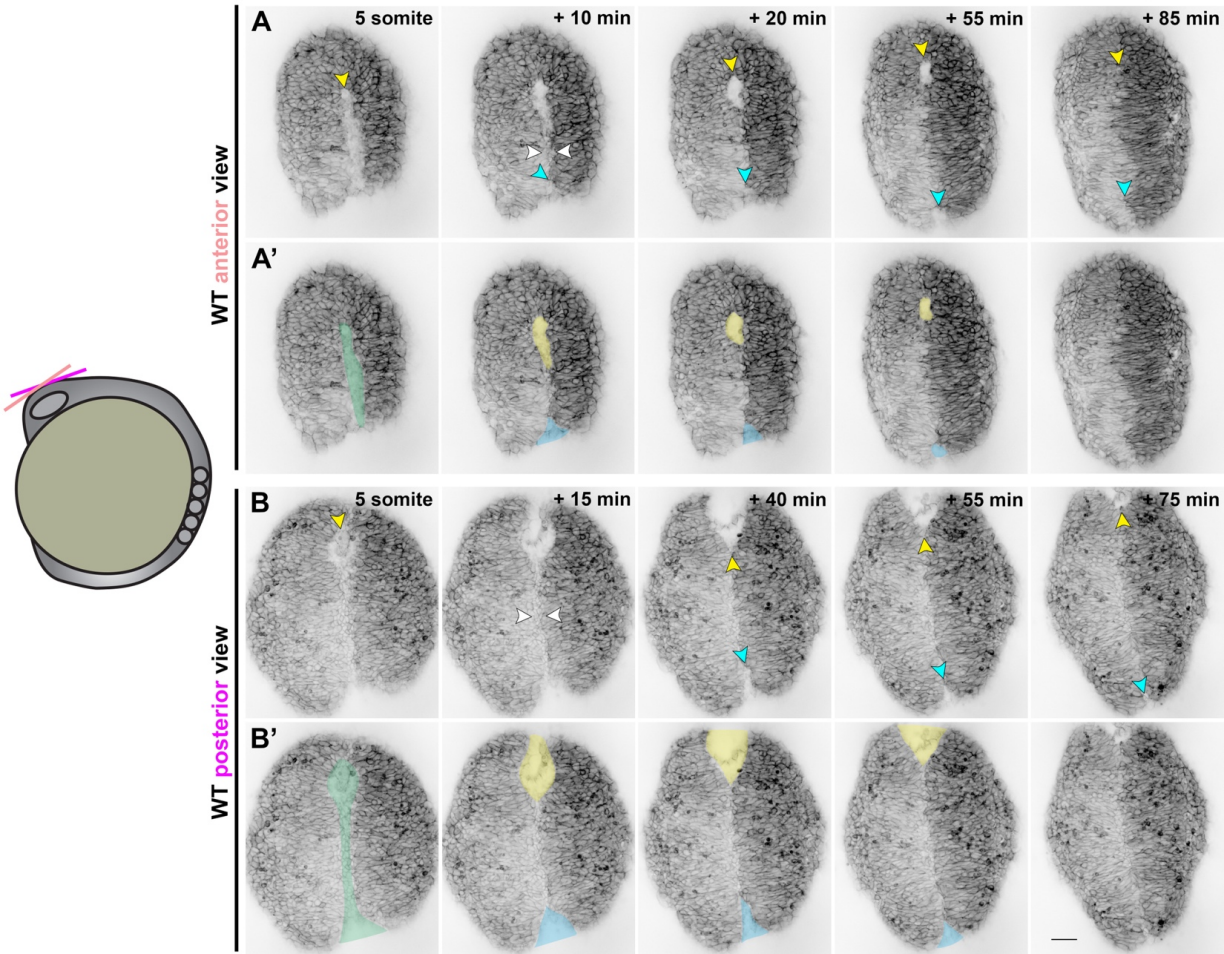
Neural fold fusion proceeds bidirectionally from a central “pinch point”. **A-B’**) Still frames from time-lapse series of anterior neural tube development in WT or *tri* sibling embryos expressing membrane GFP or mCherry beginning at the 5-somite stage, viewed dorsally from more anterior (A-A’) or posterior (B-B’) positions. **A-B**) Yellow arrowheads indicate the anterior edge of the neural groove and eventually the eye-shaped opening. White arrowheads indicate the pinch-point at which the bilateral neural folds make contact. Cyan arrowheads indicate the posterior opening that zippers closed in the posterior direction from the pinch-point. **A’-B’**) Pseudo-colored versions of the images shown in (A-B). Green indicates the early neural groove before formation of the pinch-point. Thereafter, yellow and blue indicate the anterior and posterior openings, respectively. Each image series is a single Z plane from a confocal stack and is representative of 23 individual WT and sibling embryos imaged in 6 independent trials. Additional examples are shown in Supp. Fig. 3 and Supp. videos 1-5. Anterior is up in all images, scale bar = 50 μm.

A recent study used live time-lapse imaging to directly observe closure of the forebrain neural tube in zebrafish, revealing bidirectional zippering of an eye-shaped opening between the fusing neural folds beginning at approximately 6-7 somite stage (44). To characterize neural tube closure more fully in WT embryos, we performed confocal time-lapse imaging of the anterior neural plate beginning at the 4-somite stage. In each of the 23 control embryos imaged, we observed the presence and zippering of an eye-shaped opening in the forebrain region as previously reported (44). However, examining the neural plate at earlier stages revealed a sequence of preceding morphogenetic changes (Fig. 4). At around the 5-somite stage, we observed a continuous keyhole-shaped groove in the neural plate midline with the round portion positioned anteriorly (Fig. 4A-B’, green shading). The bilateral neural folds then elevated on either side of this groove, resembling the two sides of a hot dog bun (**Supp. videos 2, 5**), and came together near the center of the keyhole to “pinch off” the anterior and posterior portions (Fig. 4B and Supp. Fig. 3, white arrows, and **Supp. videos 1-5**). Elevation of the neural folds was accompanied by apical constriction of midline neuroectoderm cells (see **Supp. video 2**), consistent with observations in the future forebrain and hindbrain (43, 44).

The posterior opening began zippering closed first, beginning at the “pinch point” and continuing posteriorly (Fig. 4A-B’, blue shading and arrows, **Supp. videos 1, 3**,), leaving the anterior portion to form the eye-shaped opening later. In some control embryos, a small opening at the posterior end of this zipper could later be seen completing closure (Fig. 4A-A’). Once the posterior opening had zippered (mostly) closed, the neural folds of the anterior portion approached one another at the midline and made contact to generate the posterior closure point of the eye-shaped opening (Fig. 4A and Supp. Fig. 3, yellow shading and arrows), as previously described (44). The anterior closure point of the eye-shaped opening arose from the anterior-most edge of the initial keyhole-shaped groove (Fig. 4 and Supp. Fig. 3, yellow arrows), and the opening zippered closed predominantly from anterior to posterior (**Supp. videos 4, 6**). As mentioned above, the posterior-most end of the posterior opening also completed its zippering at this stage. Notably, this live imaging also enabled examination of the relationship between midline-crossing C-divisions and neural tube closure. In embryos in which the left and right sides of the developing neural keel exhibited distinct levels of fluorescent protein expression (**Supp. videos 1, 3, 4, 5**), cells were only seen crossing the midline after the anterior eye-shaped opening had closed. This provides further evidence that neural fold fusion precedes C-divisions.

Together, these observations delineate a complex series of morphogenetic events that close the anterior zebrafish neural tube. First, apical constriction of midline neuroectoderm cells creates a medial hinge point and elevates the bilateral neural folds, producing a shallow groove along the dorsal midline. The neural folds come together near the center of this groove, pinching it into anterior and posterior segments. The neural folds zipper together posteriorly from the pinch point while the neural folds continue toward the midline in the anterior portion of the groove, creating the previously described eye-shaped opening that then zippers shut between two closure points. Around the time the anterior eye-shaped opening closes, the caudal-most end of the posterior portion completes its zipper closure (see model in Fig. 7).

### Neural fold fusion is delayed in Vangl2 deficient embryos.

**Figure 5.**
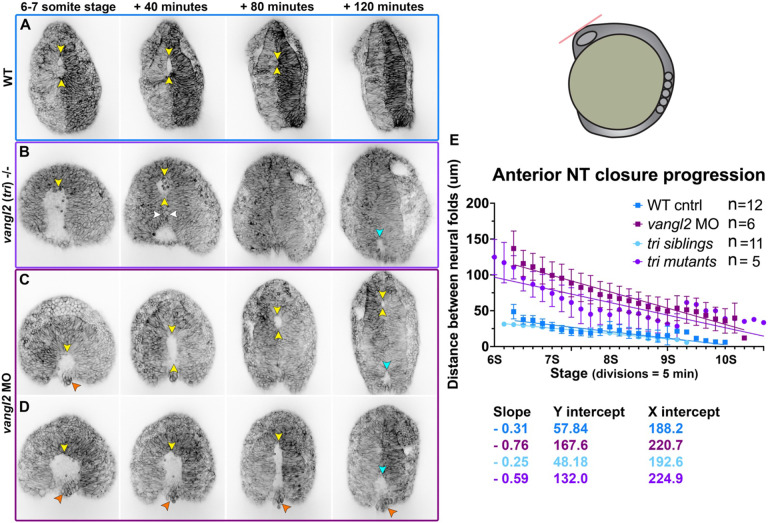
Neural fold fusion is delayed in *vangl2* deficient embryos. **A-D**) Still frames from time-lapse series of anterior neural tube development in WT (A), *tri*−/− mutant (B), and *vangl2* morphant (C) embryos expressing membrane GFP or mCherry beginning at 6-7 somite stage, viewed dorsally. Yellow arrowheads indicate the anterior edge of the eye-shaped opening, white arrowheads indicate the pinch-point, cyan arrowheads indicate the posterior opening, and orange arrowheads indicate rounded cells protruding from the neural groove of *vangl2* deficient embryos. Each image series is a single Z plane from a confocal stack and is representative of multiple embryos of that condition (see n values for each condition in E). **E**) Distance between the bilateral neural folds over time in embryos of the conditions indicated, beginning when the eye-shaped opening forms around the 6-somite stage. Symbols are mean + SEM, lines are simple linear regressions, for which slopes and intercepts are provided below. n values indicate the number of embryos measured of each condition from 4 independent *vangl2* MO and 2 independent *tri* mutant trials. Anterior is up in all images, scale bar = 50 μm. See also Supp. videos 6-7.

To determine if the DONuT is a consequence of delayed, abnormal, or failed neural fold zippering, we performed confocal time-lapse imaging of the anterior neural plate in *vangl2* deficient embryos beginning at the 4-somite stage (Fig. 5**, Supp. video 7**). We observed that the bilateral neural folds of *vangl2* morphant and *vangl2/tri*−/− embryos began much farther apart than control siblings, which led to a delay in formation of the anterior eye-shaped opening. Indeed, while control embryos had formed the eye-shaped opening by 6-7 somite stage (Fig. 5A), the neural folds of stage-matched *vangl2* mutants and morphants had not yet made contact, leaving wide gaps between them that were open to the posterior (Fig. 5B-D). This is reflected in quantitative measurements of the distance between the neural folds over time, beginning at 6-somite stage when the eye-shaped opening had formed in control embryos. A simple linear regression revealed that the distance between the neural folds of both *vangl2* morphants and mutants started nearly three times larger (Y intercepts of 167.6 and 132 μm, respectively) than sibling control embryos (Y intercepts of 57.8 and 48.2) (Fig. 5E). Interestingly, the rate of neural fold convergence was significantly higher in *vangl2* morphants and mutants (with slopes of −0.76 and −0.59, respectively) compared with their sibling controls (slopes of −0.31 and −0.25) (Fig. 5E). This accelerated closure could not fully compensate for the increased width of their neural folds, however, and closure of the anterior opening was significantly delayed in *vangl2* morphants and mutants (with X intercepts at 220.7 and 224.9 minutes, respectively) with respect to sibling controls (X intercepts of 188.2 and 192.6 minutes) (Fig. 5E).

Additional differences in neural fold fusion were apparent from these time-lapse series. First, the anterior eye-shaped opening of control embryos closed predominantly from anterior to posterior (**Supp. videos 4, 6**), whereas *vangl2* morphant and mutant neural folds zippered closed from posterior to anterior (**Supp. video 7**). The continuous nature of the anterior and posterior openings in the neural plate was also more apparent in *vangl2* deficient embryos, where “pinching” at the center of the keyhole-shaped groove was more dramatic (Fig. 5B, white arrows). Closure of the posterior opening was also substantially delayed and sometimes blocked in *vangl2* mutants and morphants. While the posterior opening was only briefly visible in control embryos after 6-somite stage and zippered closed rapidly, a large and persistent opening could be seen in the posterior region of essentially all *vangl2* morphants and mutants examined and often had not closed by the end of the imaging period at 10-somite stage (Fig. 5B-D, blue arrows). Finally, rounded cells were seen protruding from the neural groove during closure (Fig. 5B-D, orange arrows), as observed in 3D reconstructions of fixed *vangl2* morphants (Fig. 2G). These results highlight severe and regionally distinct defects in neural fold fusion in the absence of Vangl2. Because the zippering process itself was not disrupted in morphant and mutant embryos, we suspect that delayed neural fold fusion is largely the consequence of increased width of the neural plate that ultimately results from reduced CE (Fig. 3).

### The Forebrain neural folds fuse to enclose a lumen.

**Figure 6.**
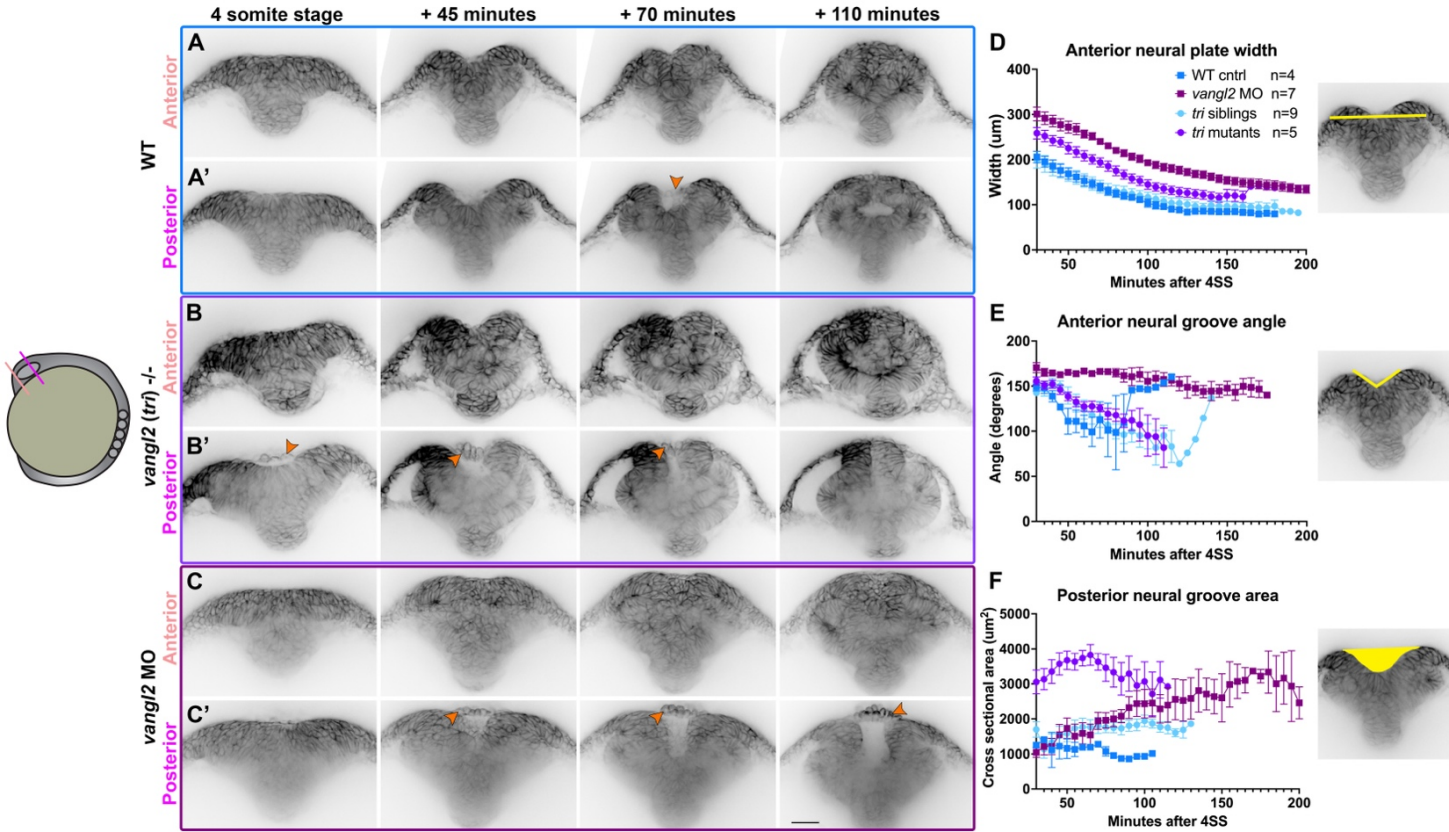
The bilateral neural folds fuse dorsally to enclose a lumen in WT, but not *vangl2* deficient embryos. **A-C’**) Still frames from time-lapse series of neural fold fusion in WT (A), *tri*−/− mutant (B), and *vangl2* morphant (C) embryos expressing membrane GFP or mCherry beginning at the 4-somite stage, viewed in transverse optical section through anterior (A-C) or posterior (A’-C’) regions of the brain. Orange arrowheads indicate periderm cells spanning the neural groove. Each image series is a single representative Z plane from a confocal stack. **D-F**) Measurements of neural plate width (D) and neural groove angle (E) within the anterior brain region and cross-sectional area of the neural groove (F) within the posterior brain region in embryos of the conditions indicated, beginning at 4-somite stage. Symbols are mean + SEM, n values indicate the number of embryos measured of each condition from 2 independent *vangl2* MO and 2 independent *tri* mutant trials. Images to the right are illustrative of the measurements made. Dorsal is up in all images, scale bar = 50 μm. See also Supp. videos 8-9.

Neural tube closure in zebrafish and amniote embryos involves not only CE and zippering, but also formation of medial and dorsolateral hinge points within the neural plate (25, 26, 29, 44, 86). Using time-lapse confocal microscopy to collect optical transverse sections through the developing forebrain region, we observed hinge point formation and neural fold elevation in WT embryos beginning at the 3-4 somite stage. The neural plate began largely flat across the apical surface but developed a prominent medial hinge point by the 5-somite stage (Fig. 6A-A’)(as described in (44)). In the anterior region of the forebrain, cells lining the V-shaped neural groove sealed up progressively from ventral to dorsal until the neural tube was closed and smooth across its outer surface (Fig. 6A). This is apparent from measurements of medial hinge point angle, which became more acute as the neural folds elevated and then widened again as the folds sealed up (Fig. 6E). Optical sections through a more posterior region of the forebrain, however, showed the bilateral neural folds elevating around a larger U-shaped groove and then fused at the dorsal side to enclose a lumen (Fig. 6A’**, Supp. video 8**). This is strikingly similar to the mechanisms of primary neurulation in amniote embryos and distinct from those of spinal cord development in zebrafish, in which the neural tube lumen is formed through cavitation of the solid neural rod (33, 40). We also observed that at this more posterior position, the periderm separated slightly from the underlying ectoderm and bridged the gap between the bilateral neural folds until they fused dorsally (Fig. 6A’, orange arrow), which can also be observed in time-lapse series from a previous study (44). These data directly demonstrate that neural folds within the forebrain region of zebrafish embryos elevate and fuse to enclose a lumen, as in many other vertebrate species.

### Neural groove formation is abnormal in *vangl2* deficient embryos.

Our live confocal imaging revealed significant delays in neural fold fusion in *vangl2* deficient embryos, but it was unclear whether this delay alone underlies DONuT formation. First, control embryos almost never exhibited a DONuT even prior to fusion of the pineal precursors and neural folds. Second, some developmentally advanced (8-somite stage) *vangl2* deficient embryos exhibited bifurcated pineal precursors in the absence of a DONuT (Fig. 2). Together, this implies that a DONuT is not simply the consequence of un-fused pineal domains but likely reflects additional developmental abnormalities. We therefore collected transverse optical sections through the developing brain of *vangl2* mutant and morphant embryos.

The anterior forebrain regions of *vangl2/ tri*−/− embryos (Fig. 6B) were wider than their siblings throughout neural tube development (Fig. 6D) but exhibited formation of a V-shaped groove that sealed from ventral to dorsal, similar to sibling controls (Fig. 6E). The posterior forebrain also exhibited a U-shaped groove with bilateral neural folds that fused dorsally, although no open lumen was apparent upon fusion (Fig. 6B’). This groove was substantially larger by cross-sectional area than sibling controls (Fig. 6F), likely due to increased width of the neural plate. Consistent with all other data in this study, *vangl2* morphants presented with a more severe phenotype than mutants. The anterior forebrain regions of morphants were even wider than mutants (Fig. 6D) and exhibited neither hinge points nor V-shaped grooves, instead resembling a solid mass of cells at this level (Fig. 6C, E). A large U-shaped groove was apparent in a more posterior region, but fusion of these folds was significantly delayed and sometimes blocked (Fig. 6C’), as evidenced by the enlarged cross-sectional area of the neural groove over time (Fig. 6F). Notably, cross-sectional area of the neural groove was larger in *vangl2* mutants than morphants at early stages (Fig. 6F), which likely reflects the combination of widened neural plates and robust hinge point formation in mutants. In both *vangl2* mutants and morphants, the periderm spanned the gap between neural folds in the posterior region as in control embryos, although this cell layer separated from the underlying neural plate earlier and by a larger distance than in controls. These cells were also highly rounded and protruded outward from the neural groove (Fig. 6B’-C’, orange arrows), indicating that cells observed protruding from the neural tube of fixed and live *vangl2* deficient embryos (Figs. 2 and 5) were almost certainly periderm. These results reveal that a combination of increased neural plate width and decreased bending at hinge points produce larger neural grooves in the forebrain region of *vangl2* morphant embryos, manifesting as a DONuT.

## Discussion

Closure of the neural tube is essential for proper development of the central nervous system, and its failure leads to deadly and debilitating congenital anomalies. Primary neurulation is well described in vertebrate models including mouse, chick, and *Xenopus*, which share a core set of cellular behaviors including convergent extension of the neural plate, apical constriction at hinge points, and dorsal fusion of the bilateral neural folds. Although neurulation in zebrafish embryos differs outwardly from these other species, it is increasingly clear that many aspects of primary neurulation are conserved, including apical constriction of neural midline cells and zippering of the neural folds (40, 43, 44). In this study, we have further characterized cell and tissue behaviors driving neural tube closure in the brain region of zebrafish embryos and how these behaviors are affected by loss of the NTD risk gene *vangl2*.

### Conservation of primary neurulation

**Figure 7.**
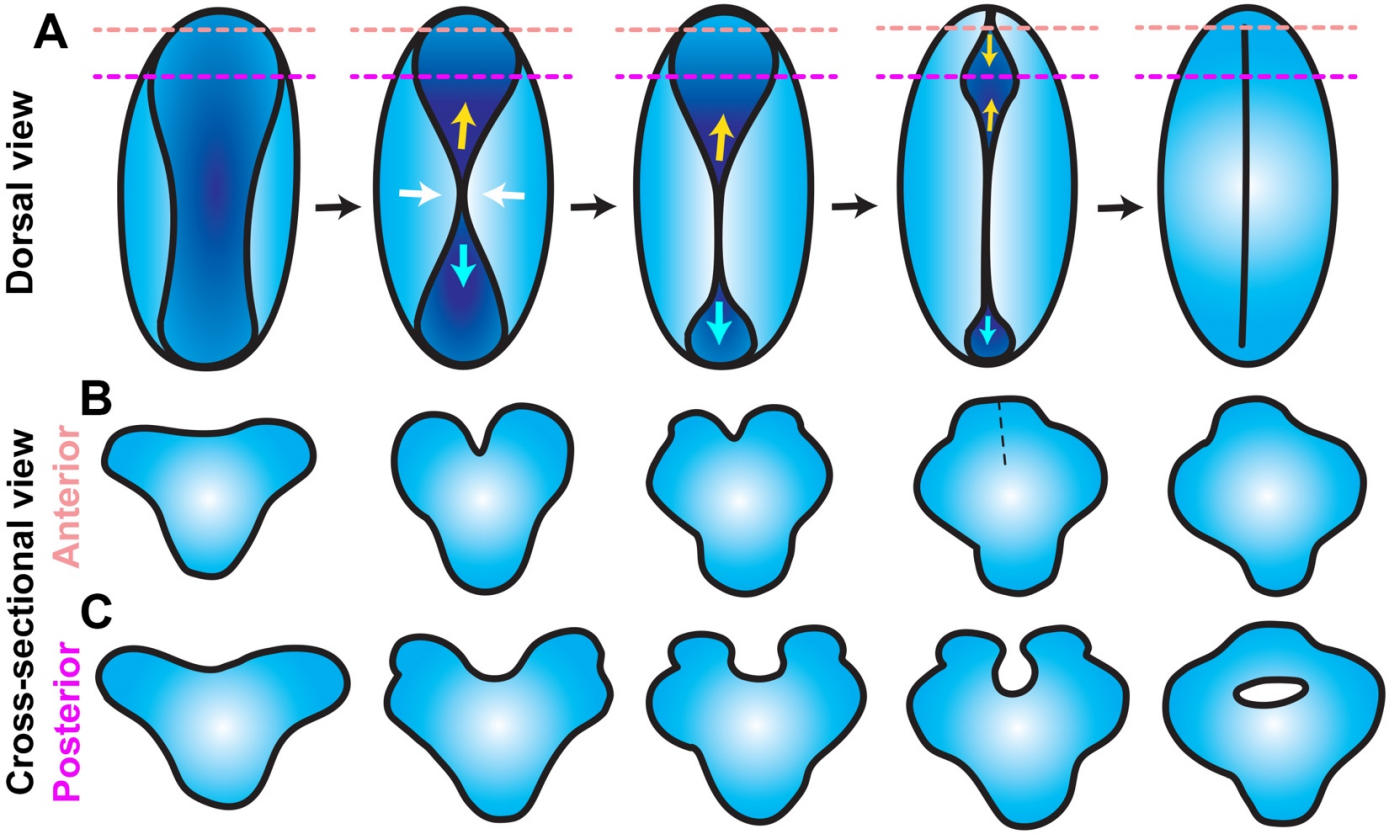
Model for anterior neural tube closure in zebrafish embryos. **A**) Diagram of the anterior (brain region) neural plate in WT zebrafish embryos from approximately 4-10 somite stage, viewed from the dorsal surface with anterior to the top. A shallow neural groove (dark blue) forms at the dorsal midline between the bilateral neural folds (light blue). The neural folds come together at a central “pinch point” (white arrows), creating anterior and posterior openings. The posterior opening zippers closed caudally from the pinch point (cyan arrows) and the anterior opening goes on to form an eye-shaped opening in the forebrain region. The anterior edge of the eye-shaped opening zippers toward the posterior while its posterior edge zippers anteriorly, closing the eye-shaped opening from both sides. The posterior opening continues to zipper toward the hindbrain until the neural folds in the entire brain region have fused. Dashed lines represent the positions of the cross-sectional views shown in (B-C). **B-C**) Cross-sectional views of anterior (B) and posterior (C) forebrain regions of the neural plate at the positions of the dashed lines in (A), dorsal is up. The anterior forebrain (B) forms a V-shaped neural groove that seals up from ventral to dorsal as the neural folds fuse. The posterior forebrain (C) forms a U-shaped neural groove between the bilateral neural folds, which approach the midline and then fuse dorsally to enclose a hollow lumen.

It has long been appreciated that the zebrafish trunk neural tube forms through in-folding, by which the lateral edges of the neural plate come together at the dorsal surface (34, 87). This is facilitated by an enrichment of myosin contractility and subsequent apical constriction of midline cells, which drives their internalization (43). Although these features are common to other vertebrate embryos, they differ in that the neural tube lumen forms in mice and chick when the bilateral neural fold enclose an empty space upon dorsal fusion, whereas the zebrafish lumen (at the level of the trunk) forms later by cavitation of a solid rod (33). Hypotheses for evolutionary drivers of this unique method of neurulation include reducing exposure of the neural tube lumen to the outside environment (88) and overcoming the high mechanical stress imposed by axial curvature of the embryo that could otherwise prevent elevation of the neural folds (89). However, our findings provide direct evidence for neural fold elevation and enclosure of a lumen in zebrafish. Our time-lapse imaging directly demonstrates that, within the future forebrain, the bilateral neural folds elevate around a midline groove then fuse at the dorsal surface, leaving a hollow lumen inside (Fig. 6, Fig. 7C). This mechanism is apparently unique to a portion of the forebrain region, as it was not observed in transverse images through more anterior (Fig. 6, Fig. 7B) or posterior regions (33, 43, 87, 90) of the neural keel, which is likely why it was not described previously. An elegant live imaging study did capture formation of hinge points and elevation of neural folds in the forebrain region (44), but did not describe lumen enclosure.

Our findings also expand our understanding of neural fold fusion within zebrafish. The aforementioned live imaging study of zebrafish forebrain (44) directly demonstrated neural fold fusion by bidirectional “zippering” of an eye-shaped opening. By imaging an earlier stage of neural development, the current study captures the events preceding formation of this closure point (Fig. 7). We first observe elevation of bilateral neural folds to create a keyhole-shaped neural groove, which then pinches together in the center to create anterior and posterior opening that zipper closed away from the pinch-point (Fig. 4). The anterior portion of the groove goes on to form the previously described eye-shaped opening (44). The posterior opening has not (to our knowledge) been described before, but a previous time-lapse imaging study of midbrain-hindbrain boundary formation captured zippering of an opening in the hindbrain (91) that we speculate is the same posterior closure point. We observed that closure at each point occurs in a reproducible order - first the central pinch-point, then the anterior followed by posterior ends of the eye-shaped opening (Fig. 7) - raising the possibility that they are analogous to the multiple discreet closure points where neural fold zippering initiates in the mouse (30, 31). It was further shown in mice that this closure is facilitated by contact between the non-neural ectoderm (NNE) (31, 92), which extends protrusions that meet across the neural groove to “button up” the neural folds (93). Our study did not directly address the behavior of NNE and provides no evidence for such protrusions, but neuroectoderm cells were previously shown to extend filopodia and make contact with cells on the contralateral side (44). We did observe, however, that the periderm overlying the developing neural tube spanned the neural groove as the neural folds elevated and fused (Fig. 6). Whether this thin epithelial sheet contributes to neural tube closure, similarly to the NNE of mouse embryos, will require additional investigation.

### Effect of vangl2 deficiency on neural development

Loss of the planar cell polarity protein and NTD risk gene *vangl2* has dramatic effects on vertebrate neural tube development. While disruption of this gene prevents neural tube closure in mice and frogs (20, 21, 56, 94), in zebrafish it produces double neural lumens divided by a mass of neuroectoderm cells that result from abnormal midline C-divisions (38, 72). By examining a more anterior region of the developing neural tube in *vangl2* deficient zebrafish embryos, we have identified additional phenotypes that more closely resemble those of other vertebrate species. During early neurulation, we observe pit-shaped openings in the future forebrain of morphant embryos (Fig. 2) that likely reflect the combination of delayed neural fold fusion and abnormal neural groove formation observed during live imaging (Figs. 5-6). We note that neural fold fusion itself is not disrupted by loss of *vangl2* (unlike mice (95)), and that zippering is actually accelerated in these embryos (Fig. 5). Instead, we find that this delay is due to increased width of the neural plate at the time of fusion and therefore, is likely a consequence of reduced CE of neuroectoderm (38, 42) (Fig. 3). Because anterior neural fold fusion is complete (even in most *vangl2* deficient embryos) by the onset of C-divisions (34, 35, 41), it is unlikely that abnormal C-divisions contribute to defects in forebrain closure. Live imaging also revealed abnormal morphology of the periderm overlying the neural plate in *vangl2* deficient embryos. Although the periderm was also seen spanning the neural folds in WT embryos (Fig. 6), it remained relatively close to the neural plate surface and its constituent cells were flat. By contrast, *vangl2* deficient periderm cells were rounded and protruded dramatically from the neural groove (Figs. 5, 6). Whether abnormal periderm cells are contributors to, a consequence of, or unrelated to delayed neural fold fusion in these embryos is unknown.

### Implications for NTD modeling in zebrafish

Zebrafish embryos are highly amenable to genetic and chemical screening techniques (96-98) that could enable identification of causative genetic variants and gene-environmental interactions, but their utility in NTD modeling has been limited by their apparent poor resemblance to mammalian neurulation. Researchers have suggested bifurcated pineal precursors as a proxy for NTDs in zebrafish embryos and have even identified gene-environment interactions that exacerbate pineal defects (45, 47). However, it was not clear to what extent these phenotypes resemble NTDs because A) they were not examined histologically and B) the mutations that induce them (in genes encoding Nodal signaling components and N-cadherin) are not associated with human NTDs. Here, we show that loss of an NTD risk gene does indeed cause widened and sometimes split pineal domains in the forebrain (Fig. 1). However, we find that this external phenotype can manifest with a variety of internal phenotypes, some of which show little resemblance to amniote NTDs (Supp. Fig. 1). By instead examining neural development at the time of neural tube closure, we avoid the confounding effects of ectopic lumen formation seen at later stages upon loss of *vangl2* or Nodal signaling (Supp. Fig. 1). Indeed, the delay in neural fold fusion in *vangl2* morphants is readily observed as a DONuT within fixed embryos at peri-closure stages, providing an easily screen-able phenotype. Whether the DONuT occurs in upon loss of other NTD risk genes, and/or whether it is common to other planar cell polarity mutants, remains to be tested. Given that open neural tubes are only apparent in the brain region of zebrafish embryos, it is also not clear whether this is a fitting model for only anterior NTDs (like anencephaly and craniorachischisis) or whether mutations causing posterior NTDs like spina bifida would yield similar phenotypes. Taken together, this study provides direct evidence for conservation of fold-and-fuse neural tube closure within zebrafish, highlighting their potential for NTD modeling.

## Materials and Methods

### Zebrafish

Adult zebrafish were maintained through established protocols (99) in compliance with the Baylor College of Medicine Institutional Animal Care and Use Committee. Embryos were obtained through natural mating and staging was based on established morphology (100). Studies were conducted using AB WT, *tdgf1/oep^tz257^(*101), *vangl2/ trilobite^vu67^* (102), and TgBAC[*flh:flh-kaede*] (82) embryos. Fish were crossed from their home tank at random and embryos were chosen for injection and inclusion in experiments at random.

### Microinjection of synthetic mRNA and morpholino oligonucleotides

Single-celled embryos were placed in agarose molds (Adaptive Science tools I-34) and injected with 0.5-2 nL volumes using pulled glass needles (Fisher Sci #50-821-984). mRNAs were transcribed using the SP6 mMessage mMachine kit (Fisher Sci #AM1340) and purified using Biorad Microbiospin columns (Biorad #7326250). Each embryo was injected with 100 pg memGFP or 200 pg mCherry mRNA, and/or 2 ng *vangl2* MO-4 ((83) sequence: 5' - AGTTCCACCTTACTCCTGAGAGAAT - 3').

### Whole mount in situ hybridization

Antisense riboprobes were transcribed using NEB T7 or T3 RNA polymerase (NEB #M0251s and Fisher #501047499) and labeled with digoxygenin (DIG) NTPs (Sigma/Millipore #11277073910). Whole mount in situ hybridization (WISH) was performed according to (103) with minor modifications. Embryos were fixed as described above, washed in PBS + 0.1% Tween-20 (PBT), gradually dehydrated, and stored in methanol at −20C. Immediately prior to staining, embryos were rehydrated into PBT and hybridized overnight with antisense probes within the wells of a 24-well plate. Embryos were gradually washed into SSC buffer and then into PBT before overnight incubation with an anti-DIG primary antibody at 1:5000 (Roche #11093274910). Embryos were washed in PBT and then staining buffer before developing in BM Purple staining solution (Roche #11442074001). Embryos were washed and stored in stop buffer (10 mM EDTA in PBT) until imaging.

### Histology

After whole mount in situ hybridization, the head regions of 28 hpf embryos were isolated, mounted in Tissue Tek O.C.T. medium (VWR #25608-930) within plastic base molds, and snap frozen in liquid nitrogen. 14 μm serial sections were cut and collected by the Baylor College of Medicine RNA In Situ Hybridization Core. Sections were mounted under coverslips and imaged using a Nikon Fi3 color camera on the Nikon ECLIPSE Ti2 microscope described below.

### Inhibitor treatments

Nodal inhibitor SB505124 (VWR #103540-834) was stored as a 10 mM stock in DMSO at 4 C. Embryos were dechorionated prior to treatment with 50 μM SB505124 in 0.3x Danieau’s solution at the stages indicated. Embryos were incubated at 28.5 C within the agarose-coated wells of a 6-well plate until 28 hpf, at which time they were fixed as described above and processed for WISH.

### Phalloidin staining

Embryos were fixed as described above, rinsed in PBT, and either stained directly or dehydrated and stored at −20 C in ethanol for later use. Embryos were rehydrated in PBS + 0.1% Triton-X (PBTr) immediately before staining and incubated with Alexa Fluor 546 Phalloidin (ThermoFisher A22283) in PBTr for several hours. Embryos were rinsed in PBTr and mounted for confocal imaging as described below.

### Microscopy

Fixed phalloidin stained embryos were mounted in 3% methylcellulose, and live embryos were mounted in 0.35% low-melt agarose (ThermoFisher #16520100) in glass bottomed 35 mm petri dishes (Fisher Sci #FB0875711YZ) prior to imaging. Confocal Z-stacks were collected using a Nikon ECLIPSE Ti2 confocal microscope equipped with a Yokogawa W1 spinning disk unit, PFS4 camera, and 405/488/561nm lasers (emission filters: 455/50, 525/36, 605/52). Confocal Z-stacks were obtained with a 1 μm (fixed embryos) or 2 μm (live embryos) step size using a Plan Apo Lambda 20X lens. For time-lapse series, embryos were maintained at 28.5 C in a Tokai Hit STX stage top incubator and Z-stacks were collected at 5-minute intervals. Images of WISH-stained embryos were taken with a Nikon Fi3 color camera on a Nikon SMZ745T stereoscope.

### Image analysis

ImageJ/FIJI was used to visualize and measure all microscopy data sets. Researchers were blinded to the conditions of all image data using the *blind_renamer* Perl script prior to analysis. Measurements of embryonic structures from fixed embryos, including pineal precursors and somites, were made by drawing a line from one side of the structure to the other at its widest point. Distance between neural folds, neural plate width, and neural grove angle and cross-sectional area were measured similarly from images of live embryos. 3D projections of confocal z-stacks were made using the ‘3D projec’ plugin.

### Statistical Analysis

Graphpad Prism 10 software was used to perform statistical analyses and to generate graphs for the data collected during image analysis. Datasets were tested for normality prior to analysis and statistical tests were chosen accordingly. The statistical tests used for each data set are noted in figure legends.

## Supplementary Material

Supplement 1

## Summary statement

The anterior neural tube of zebrafish exhibits fold-and-fuse neurulation which is disrupted upon loss of Vangl2, highlighting conservation of vertebrate neurulation and the potential to model neural tube defects in zebrafish.
