# Supplementary material for: Fold-and-fuse neurulation in zebrafish requires Vangl2
Source: bioRxiv. 2024 Dec 2:2023.11.09.566412. Originally published 2023 Nov 9. Preprint. [Version 2] doi: 10.1101/2023.11.09.566412 (PMC10659374; doi:10.1101/2023.11.09.566412)
Supplement: Supplement 1 [file NIHPP2023.11.09.566412v2-supplement-1.pdf]

bioRxiv preprint doi: https://doi.org/10.1101/2023.11.09.566412; this version posted December 2, 2024. The copyright holder for this preprint (which was not certified by peer review) is the author/funder, who has granted bioRxiv a license to display the preprint in perpetuity. It is made available under aCC-BY 4.0 International license.

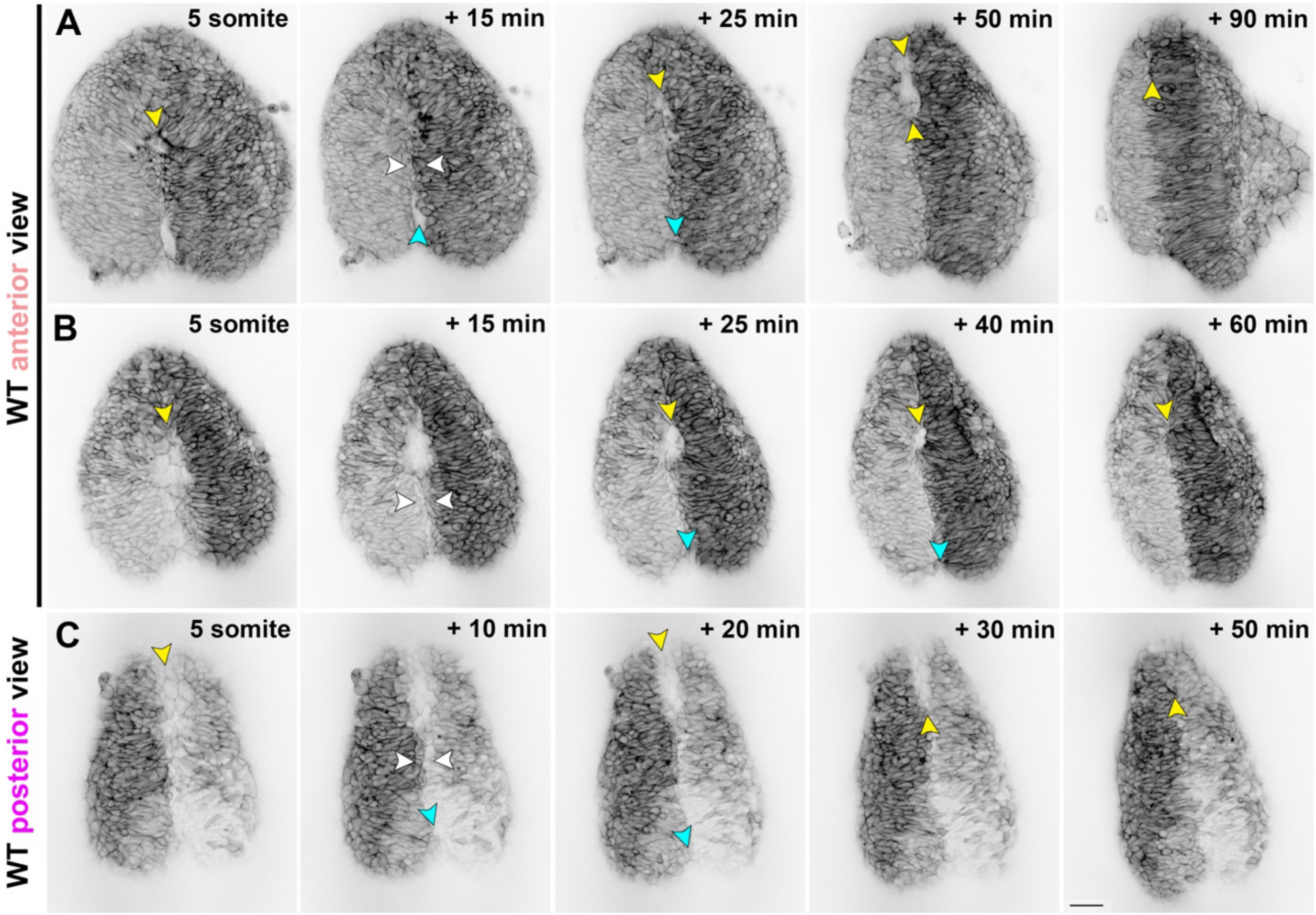

Supplemental Figure 1. Live imaging reveals neural fold fusion dynamics in live WT embryos. **A-C**) Still frames from time-lapse series of anterior neural tube development in WT or *tri* sibling embryos expressing membrane GFP or mCherry beginning at the 5 somite stage, viewed dorsally from more anterior (A-B) or posterior (C) positions. Yellow arrowheads indicate the anterior edge of the neural groove and eventually the eye-shaped opening. White arrowheads indicate the pinch point at which the bilateral neural folds make contact. Cyan arrowheads indicate the posterior opening that zippers closed in the posterior direction from the pinch point. Anterior is up in all images, scale bar = 50 μm.

bioRxiv preprint doi: https://doi.org/10.1101/2023.11.09.566412; this version posted December 2, 2024. The copyright holder for this preprint (which was not certified by peer review) is the author/funder, who has granted bioRxiv a license to display the preprint in perpetuity. It is made available under aCC-BY 4.0 International license.

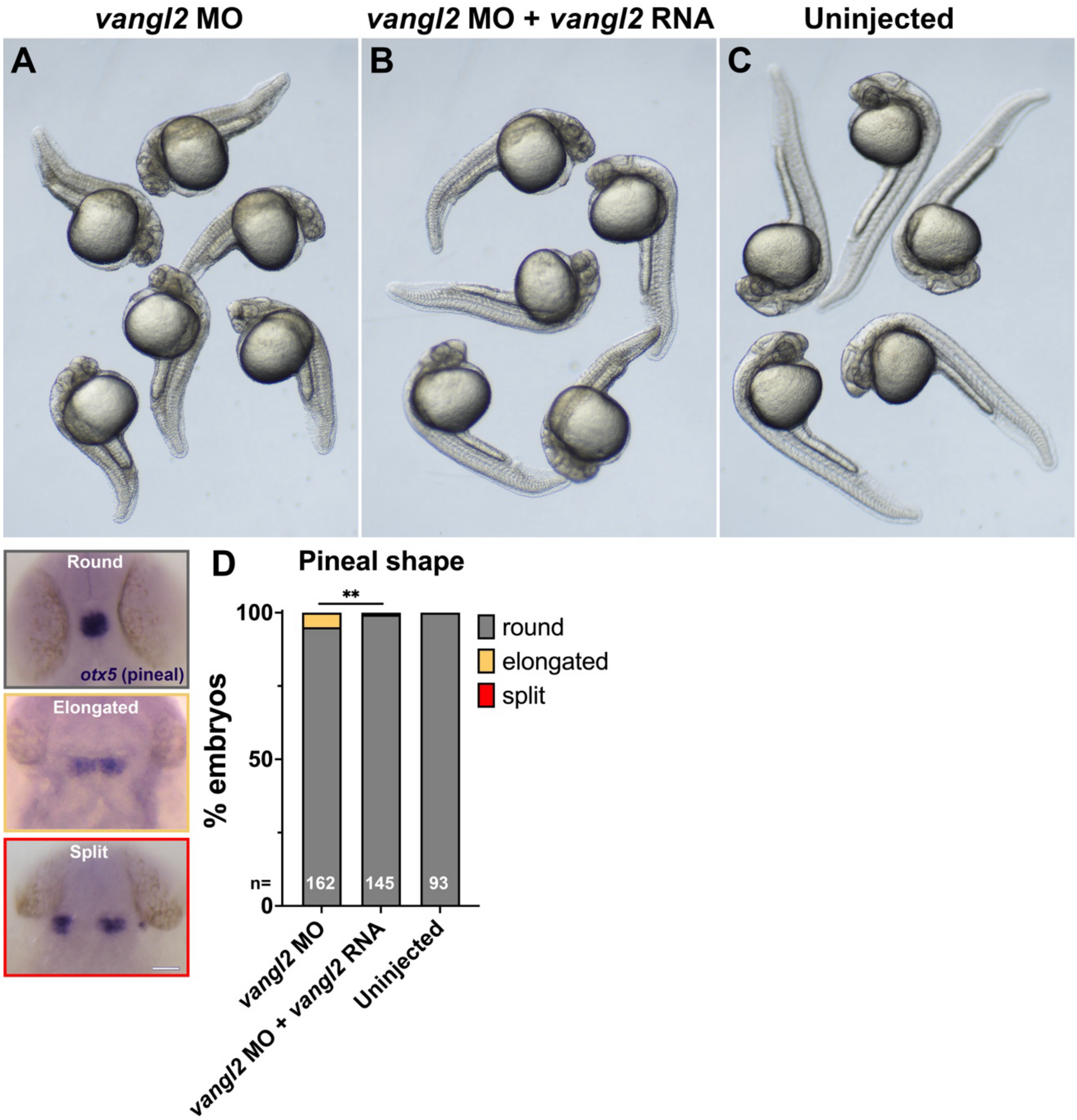

Supplemental Figure 2. ***vangl2*** **mRNA largely rescues** ***vangl2*** **morpholino day 1 phenotypes.**
A-C) Live embryos at approximately 28 hpf injected at the 1-cell stage with 2 ng *vangl2* morpholino (A), 2 ng *vangl2* morpholino + 10 pg *vangl2* mRNA lacking the MO-binding site (B), or uninjected siblings. Images are representative of 3 independent trials. **D**) Classification of pineal shape in 28 hpf control, *vangl2* morphant, and rescued morphant embryos WISH stained for *otx5*. n values indicate the number of embryos of each condition measured from 3 independent trials. **p=0.0015, Fisher's exact test.

bioRxiv preprint doi: https://doi.org/10.1101/2023.11.09.566412; this version posted December 2, 2024. The copyright holder for this preprint (which was not certified by peer review) is the author/funder, who has granted bioRxiv a license to display the preprint in perpetuity. It is made available under aCC-BY 4.0 International license.

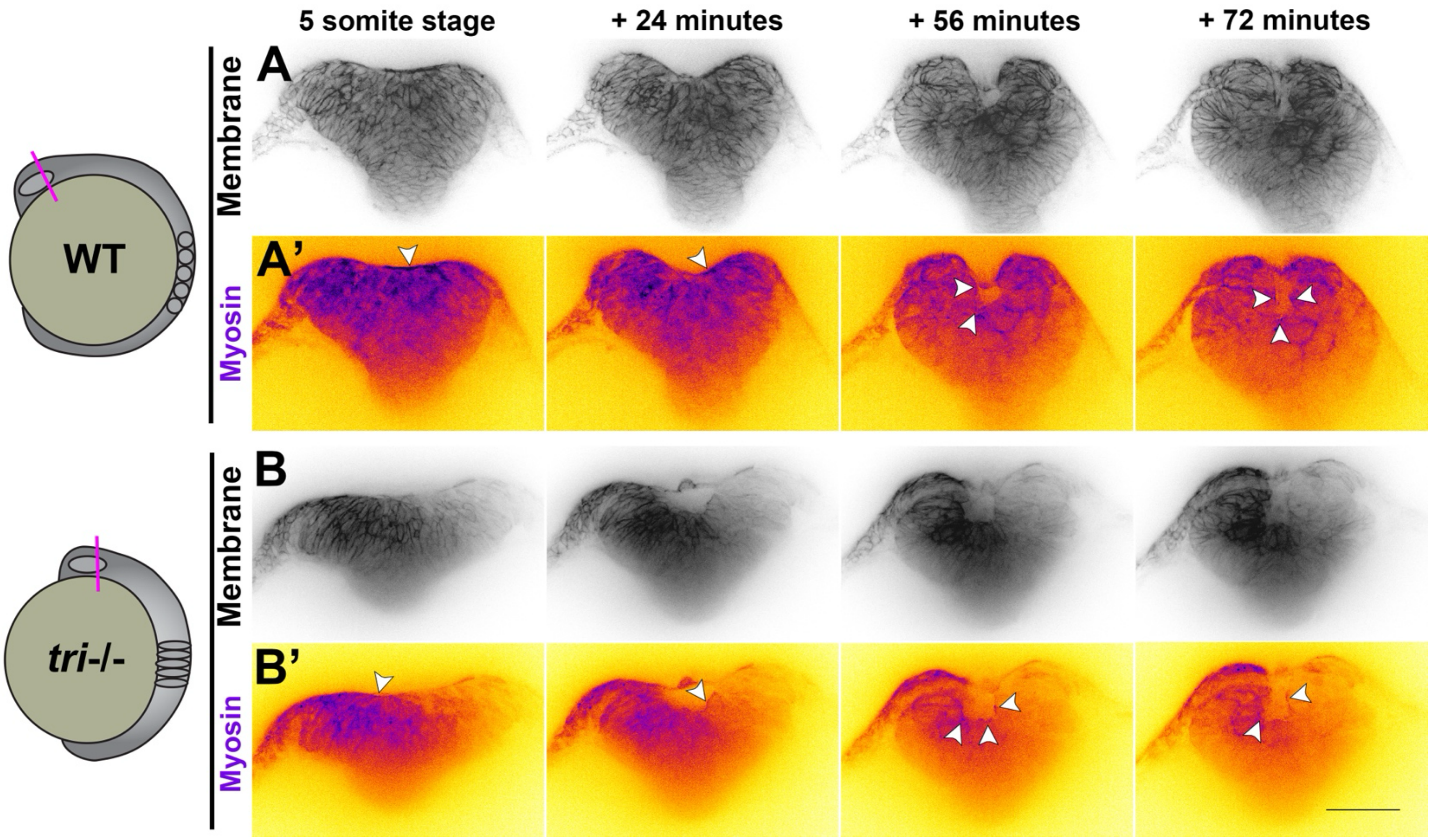

Supplemental Figure 3. Myosin accumulates apically within the neural groove of WT and *vangl2/ tri-/-* mutant embryos.

A-B') Still frames from time-lapse series of neural fold fusion in WT (A) and *tri*-/- mutant (B) embryos expressing membrane mCherry (A-B, black) and Sf9-mNeon (A'-B', inverted Fire look up table) beginning at the 5-somite stage, viewed in transverse optical section through the posterior region of the forebrain. Arrowheads indicate Myosin localization to the apical surfaces of cells comprising the neural groove. Each image series is a single Z plane from a confocal stack representative of 4 sibling and 4 mutant Sf9-expressing embryos. Dorsal is up in all images, scale bar = 100 μm.

bioRxiv preprint doi: https://doi.org/10.1101/2023.11.09.566412; this version posted December 2, 2024. The copyright holder for this preprint (which was not certified by peer review) is the author/funder, who has granted bioRxiv a license to display the preprint in perpetuity. It is made available under aCC-BY 4.0 International license.

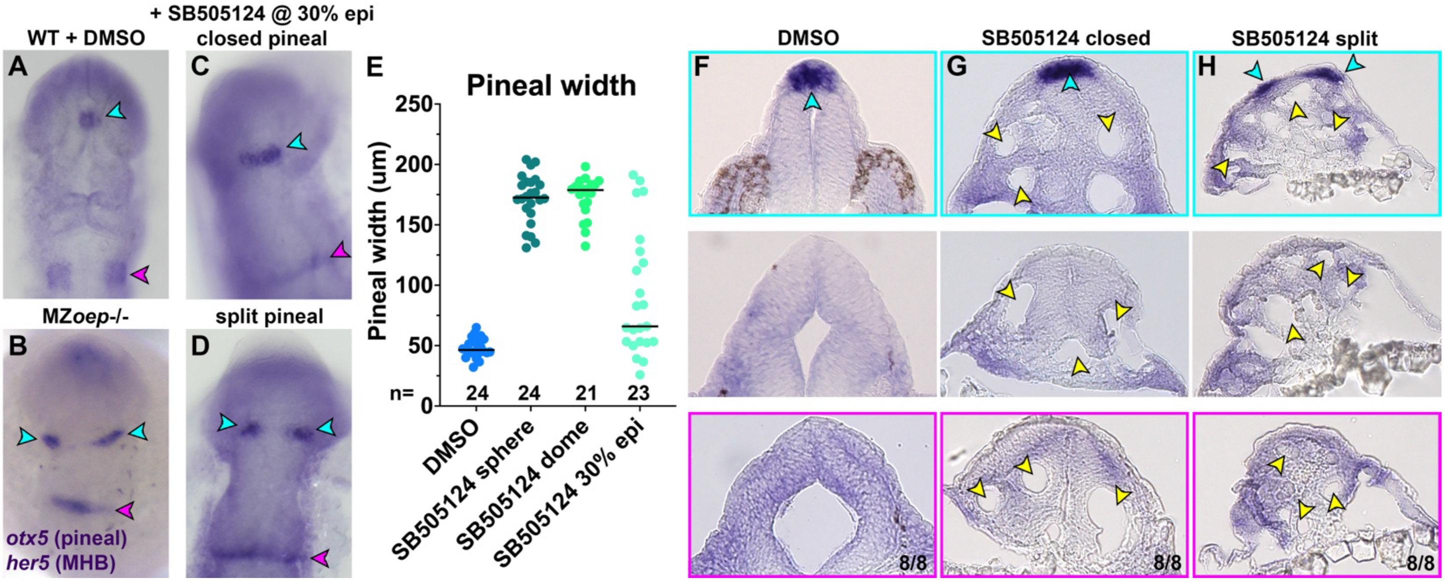

Supplemental Figure 4. Distinct neural tube morphologies underlie split pineal phenotypes in Nodal deficient embryos.

A-D) Representative images of the anterior neural tube in DMSO- treated WT (A), MZ*oep*-/-, or SB505124-treated (C-D) embryos at 28 hpf WISH stained for *otx5* and *her5*, viewed dorsally. Cyan arrowheads indicate pineal precursors, magenta arrowheads indicate the MHB. **E**) Width of pineal precursor domains in embryos of the conditions indicated, measured from *otx5* WISH at 28 hpf (as shown in A-D). Each dot represents a single embryo, black bars are median values. **F-H**) Transverse histological sections through the anterior neural tube at the level of the epithalamus (top panels), midbrain (middle panels), and MHB (bottom panels) in 28 hpf embryos of the conditions indicated. Cyan arrowheads indicate pineal precursors stained by *otx5* WISH. Yellow arrowheads indicate ectopic lumens in a Swiss cheese-like pattern. Fractions indicate the number of embryos with the depicted phenotype over the total number of embryos examined for each condition. Anterior is up in (A-D), dorsal is up in (F-H).

bioRxiv preprint doi: https://doi.org/10.1101/2023.11.09.566412; this version posted December 2, 2024. The copyright holder for this preprint (which was not certified by peer review) is the author/funder, who has granted bioRxiv a license to display the preprint in perpetuity. It is made available under aCC-BY 4.0 International license.

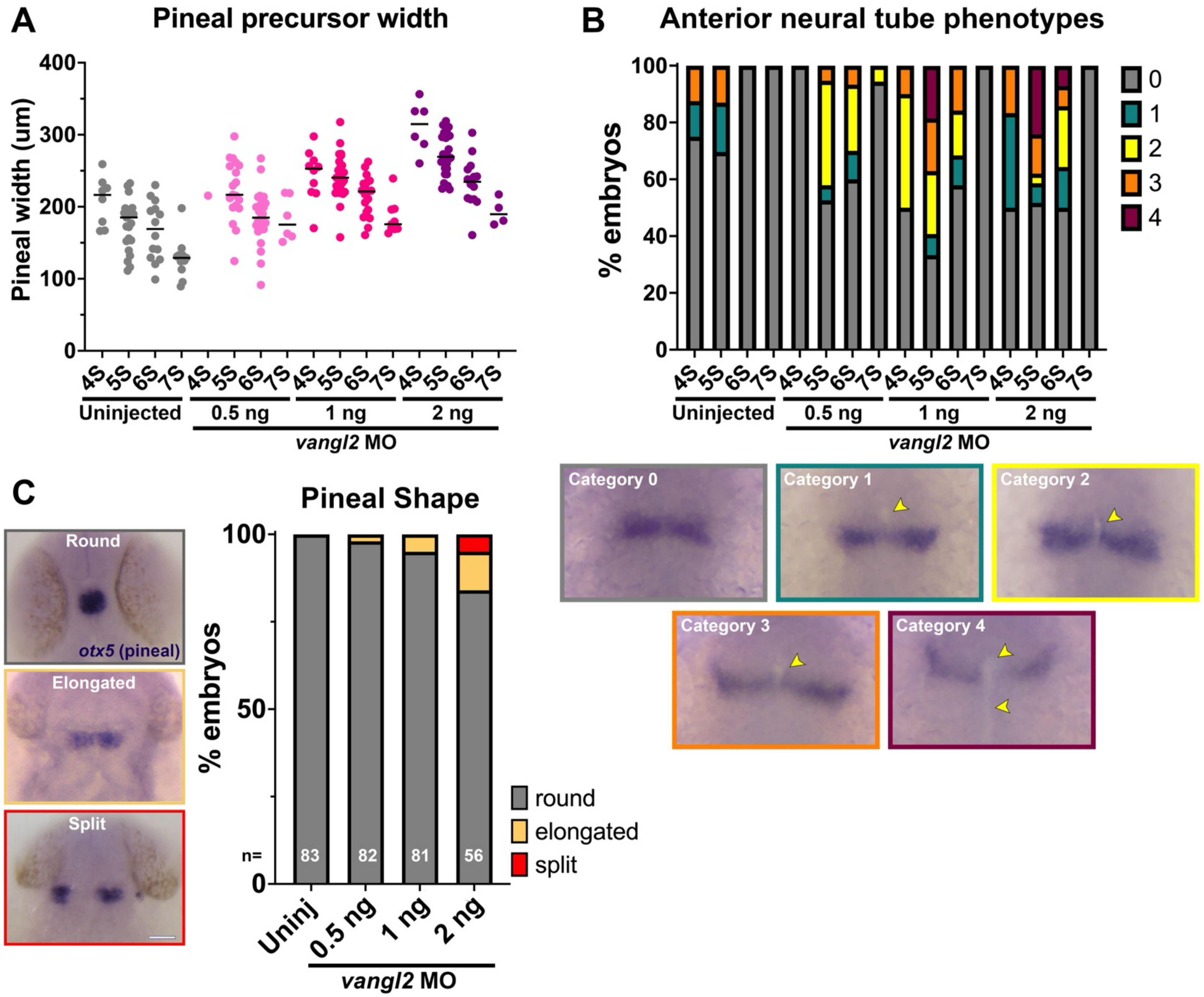

Supplemental Figure 5. Severity of neural tube phenotypes increases with dose of *vangl2* MO.
A) Width of pineal precursor domains in embryos injected with increasing doses of *vangl2* morpholino and uninjected controls at the stages indicated. Each dot represents a single embryo from 3 independent trials, black bars are median values. **B**) Percentage of embryos injected with each dose of *vangl2* morpholino at the stages indicated exhibiting the categories of anterior neural tube phenotypes shown below. **C**) Classification of pineal shape in 28 hpf control and *vangl2* MO-injected embryos WISH stained for *otx5*. n values indicate the number of embryos of each condition measured from 3 independent trials. ***p=0.0004, Fisher's exact test. Anterior is up in all images.

bioRxiv preprint doi: https://doi.org/10.1101/2023.11.09.566412; this version posted December 2, 2024. The copyright holder for this preprint (which was not certified by peer review) is the author/funder, who has granted bioRxiv a license to display the preprint in perpetuity. It is made available under aCC-BY 4.0 International license.

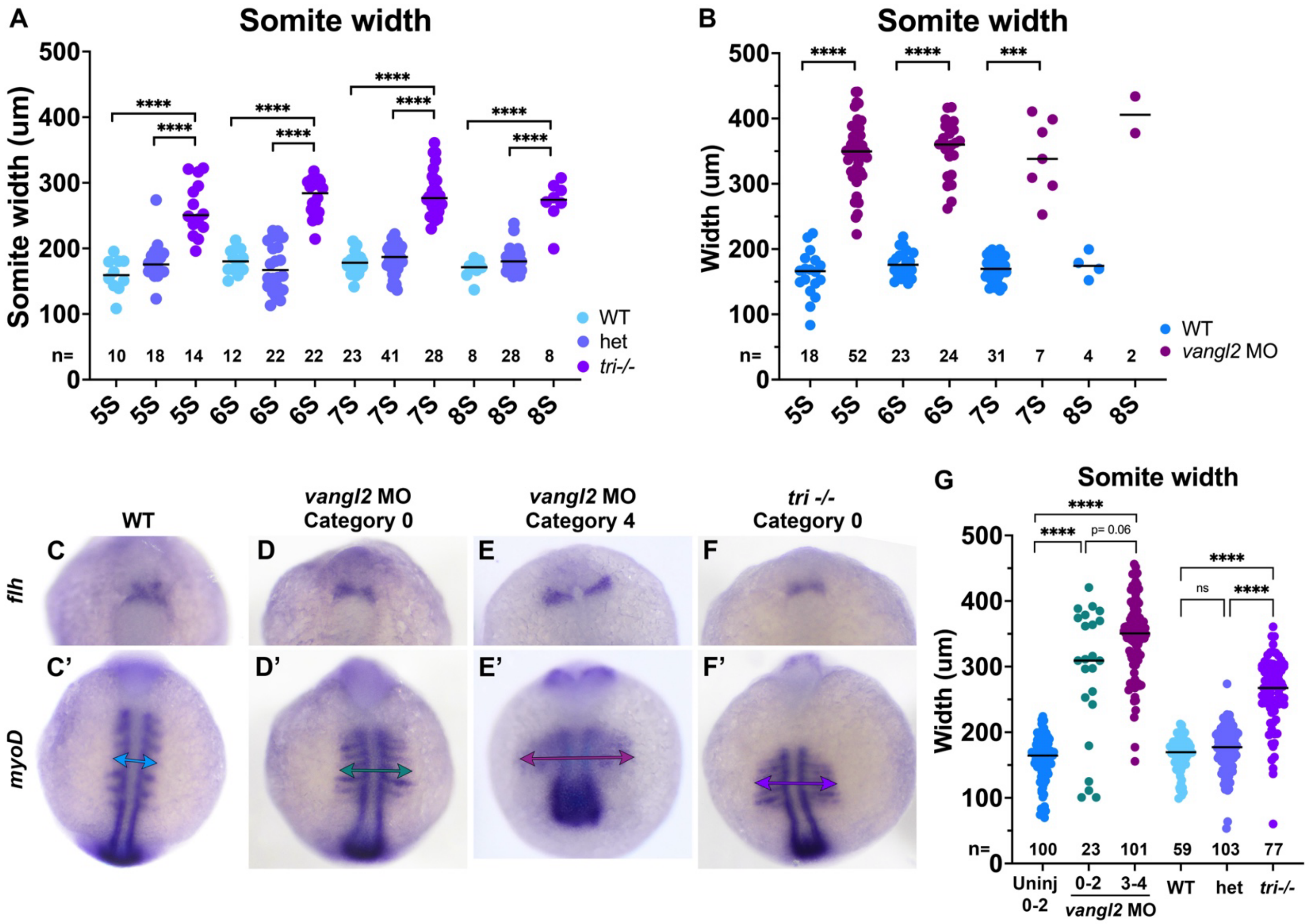

Supplemental Figure 6. Anterior neural plate phenotypes are correlated with somite width.
A-B) Width of *myoD*+ somites in *tri*+/- incross (A) and *vangl2* morphant and control (B) embryos at the stages indicated. ****p<0.0001, Welch's ANOVA with Dunnett's multiple comparisons (A), ****p<0.0001, ***p=0.0002, multiple T-tests with Welch's correction (B). **C-F'**) Representative images of pineal precursors (*flh* WISH) and somites/adaxial cells (*myoD* WISH) in embryos of the condition and with the category of neural plate phenotype indicated at the 7-somite stage. Double arrows indicate somite width. **G**) Somite width (as shown in C'-F') in embryos of the conditions indicated at 3-7 somite stages, including *vangl2* morphant embryos with category 0-2 (teal) or category 3-4 (burgundy) neural tube phenotypes. Each dot represents a single embryo, black bars are median values. ****p<0.0001, Kruskal-Wallis test with Dunn's multiple comparisons. n values indicate the number of embryos of each stage/condition measured from 3 independent trials. Anterior is up in all images.

bioRxiv preprint doi: https://doi.org/10.1101/2023.11.09.566412; this version posted December 2, 2024. The copyright holder for this preprint (which was not certified by peer review) is the author/funder, who has granted bioRxiv a license to display the preprint in perpetuity. It is made available under aCC-BY 4.0 International license.

Supplemental video legends

Supp. video 1: Posterior view of neural groove formation in the forebrain region of a wild-type zebrafish embryo labeled with membrane-GFP. Movie begins at approximately the 4 somite stage and each frame = 5 minutes. Video shows a single Z plane of a 3D confocal time series.

Supp. video 2: Anterior view of neural fold fusion in the forebrain region of a wild-type zebrafish embryo labeled with membrane-GFP. Movie begins at the 4-5 somite stage and each frame = 5 minutes. Video shows a single Z plane of a 3D confocal time series.

Supp. video 3: Anterior view of neural groove formation in the forebrain region of a wild-type zebrafish embryo labeled with membrane-mCherry and the Sf9-mNeon Myosin reporter. Movie begins at approximately 3 somite stage and each frame = 5 minutes. The left and right panels show a single Z plane at deeper and more superficial positions in the Z-stack, respectively.

Supp. video 4: Anterior view of neural fold fusion in the forebrain region of a wild-type zebrafish embryo labeled membrane-mCherry and the Sf9-mNeon Myosin reporter. Movie begins at the 4-5 somite stage and each frame = 5 minutes. Video shows a single Z plane of a 3D confocal time series.

Supp. video 5: Neural fold fusion in the forebrain region of a wild-type zebrafish embryo labeled with membrane-GFP. Movie begins at the 6-7 somite stage and each frame = 5 minutes. Video shows a single Z plane of a 3D confocal time series.

Supp. video 6: Posterior view of neural groove formation in the forebrain region of a *tri*-/- mutant zebrafish embryo labeled with membrane-mCherry. Movie begins at the 6-7 stage and each frame = 5 minutes. Video shows a single Z plane of a 3D confocal time series.

Supp. video 7: Posterior view of neural fold fusion in the forebrain region of a *tri*-/- mutant zebrafish embryo labeled membrane-mCherry and the Sf9-mNeon Myosin reporter. Movie begins at the 6-7 somite stage and each frame = 5 minutes. Video shows a single Z plane of a 3D confocal time series.

Supp. video 8: Neural fold fusion in the forebrain region of a *vangl2* morphant zebrafish embryo labeled with membrane-GFP. Movie begins at the 6-7 somite stage and each frame = 5 minutes. Video shows a single Z plane of a 3D confocal time series.

Supp. video 9: Optical transverse section through the posterior forebrain region of a wild-type zebrafish embryo labeled with membrane-GFP. Movie begins at the 4 somite stage and each frame = 5 minutes. Video shows a single Z plane of a 3D confocal time series.

bioRxiv preprint doi: https://doi.org/10.1101/2023.11.09.566412; this version posted December 2, 2024. The copyright holder for this preprint (which was not certified by peer review) is the author/funder, who has granted bioRxiv a license to display the preprint in perpetuity. It is made available under aCC-BY 4.0 International license.

Supp. video 10: Optical transverse section through the posterior forebrain region of a *tri*-/- mutant zebrafish embryo labeled with membrane-GFP. Movie begins at the 4 somite stage and each frame = 5 minutes. Video shows a single Z plane of a 3D confocal time series.

Supp. video 11: Optical transverse section through the posterior forebrain region of a *vangl2* morphant zebrafish embryo labeled with membrane-GFP. Movie begins at the 4 somite stage and each frame = 5 minutes. Video shows a single Z plane of a 3D confocal time series.
