# Supplementary material for: Vangl2 deficient zebrafish exhibit hallmarks of neural tube closure defects
Source: bioRxiv. 2023 Nov 9:2023.11.09.566412. Preprint. [Version 1] doi: 10.1101/2023.11.09.566412 (PMC10659374; doi:10.1101/2023.11.09.566412)
Supplement: Supplementary file 1 [file NIHPP2023.11.09.566412v1-supplement-1.pdf]

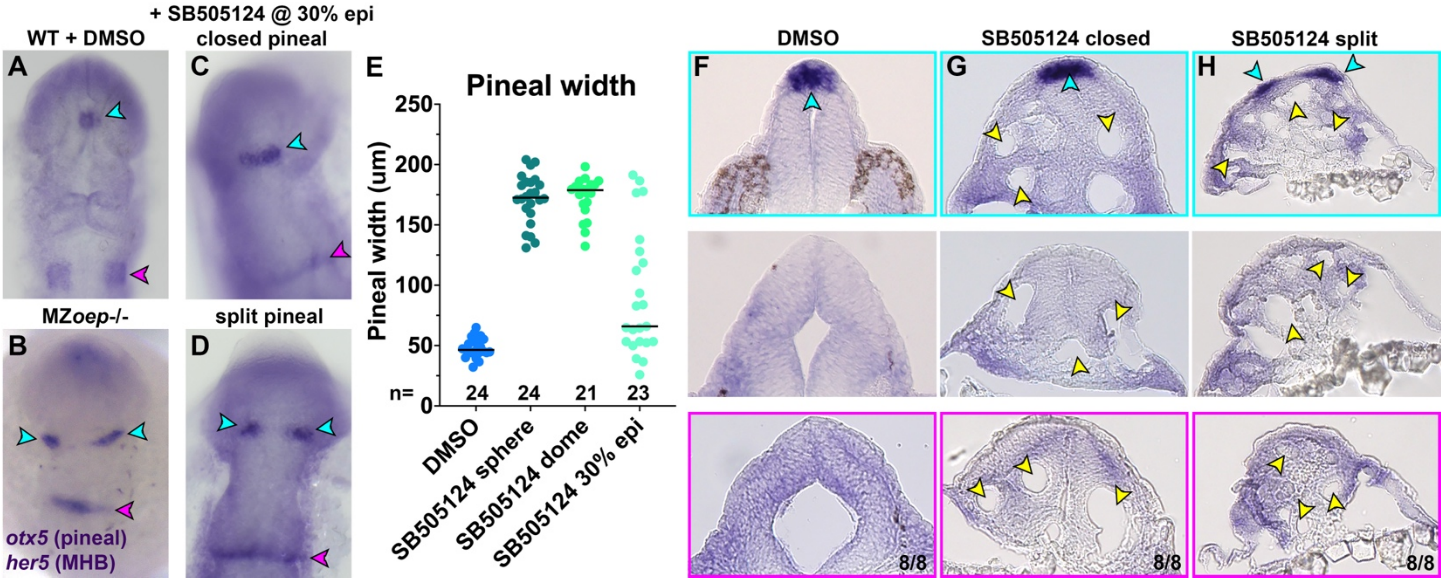

Supplemental Figure 1. Distinct neural tube morphologies underlie split pineal phenotypes in Nodal deficient embryos.

A-D) Representative images of the anterior neural tube in DMSO- treated WT (A), MZ*oep*-/-, or SB505124-treated (C-D) embryos at 28 hpf WISH stained for *otx5* and *her5*, viewed dorsally. Cyan arrowheads indicate pineal precursors, magenta arrowheads indicate the MHB. **E**) Width of pineal precursor domains in embryos of the conditions indicated, measured from *otx5* WISH at 28 hpf (as shown in A-D). Each dot represents a single embryo, black bars are median values. **F-H**) Transverse histological sections through the anterior neural tube at the level of the epithalamus (top panels), midbrain (middle panels), and MHB (bottom panels) in 28 hpf embryos of the conditions indicated. Cyan arrowheads indicate pineal precursors stained by *otx5* WISH. Yellow arrowheads indicate ectopic lumens in a Swiss cheese-like pattern. Fractions indicate the number of embryos with the depicted phenotype over the total number of embryos examined for each condition. Anterior is up in (A-D), dorsal is up in (F-H).

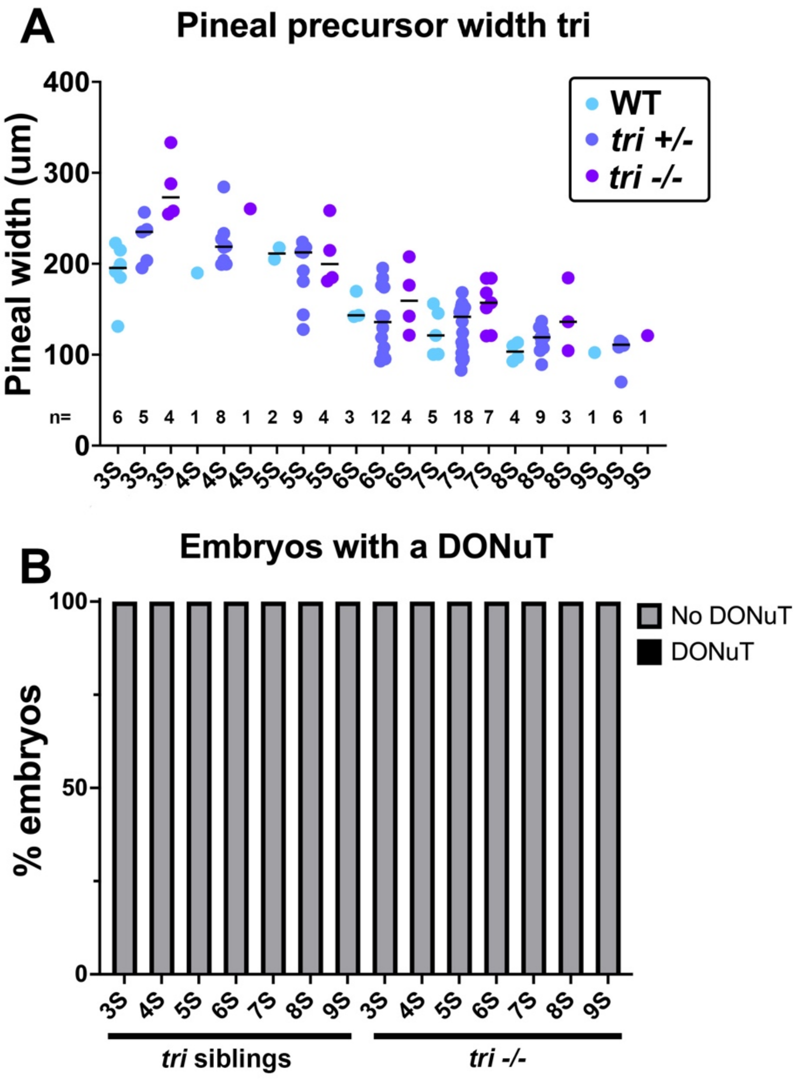

Supplemental Figure 2. Trilobite mutants do not exhibit DONuTs.
A) Width of pineal precursor domains (as shown in A-B) in WT (blue), *tri* het (indigo), and *tri* homozygous (purple) embryos at the stages indicated. Each dot represents a single embryo, black bars are median values. n values indicate the number of embryos of each stage/condition measured from 3 independent trials. **B**) Percentage of embryos from *tri* heterozygote incrosses of the stage/genotype indicated with (black) or without (gray) a DONuT.

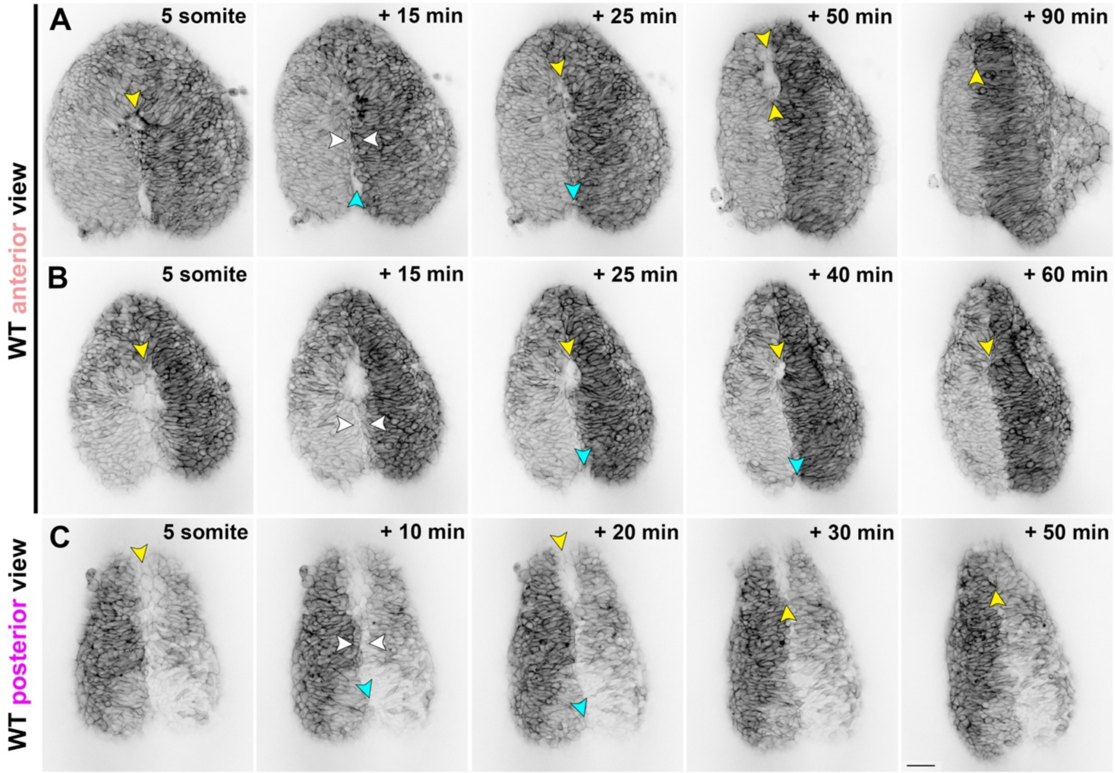

Supplemental Figure 3. Live imaging reveals neural fold fusion dynamics in live WT embryos.
A-C) Still frames from time-lapse series of anterior neural tube development in WT or *tri* sibling embryos expressing membrane GFP or mCherry beginning at the 5-somite stage, viewed dorsally from more anterior (A-B) or posterior (C) positions. Yellow arrowheads indicate the anterior edge of the neural groove and eventually the eye-shaped opening. White arrowheads indicate the pinch-point at which the bilateral neural folds make contact. Cyan arrowheads indicate the posterior opening that zippers closed in the posterior direction from the pinch-point. Anterior is up in all images, scale bar = 50 μm.

Supplemental video descriptions

Supp. video 1: Anterior view of neural fold fusion in the forebrain region of a wild-type zebrafish embryo labeled with membrane-GFP. Movie begins at the 4-5 somite stage and each frame = 5 minutes. Video shows a single Z plane of a 3D confocal time series.

Supp. video 2: Posterior view of neural fold fusion in the forebrain region of a wild-type zebrafish embryo labeled with membrane-GFP. Movie begins at the 4-5 somite stage and each frame = 5 minutes. Video shows a single Z plane of a 3D confocal time series.

Supp. video 3: Anterior view of neural fold fusion in the forebrain region of a wild-type zebrafish embryo labeled with membrane-GFP. Movie begins at the 4-5 somite stage and each frame = 5 minutes. Video shows a single Z plane of a 3D confocal time series.

Supp. video 4: Anterior view of neural fold fusion in the forebrain region of a wild-type zebrafish embryo labeled with membrane-GFP. Movie begins at the 4-5 somite stage and each frame = 5 minutes. Video shows a single Z plane of a 3D confocal time series.

Supp. video 5: Posterior view of neural fold fusion in the forebrain region of a wild-type zebrafish embryo labeled with membrane-GFP. Movie begins at the 4-5 somite stage and each frame = 5 minutes. Video shows a single Z plane of a 3D confocal time series.

Supp. video 6: Neural fold fusion in the forebrain region of a wild-type zebrafish embryo labeled with membrane-GFP. Movie begins at the 6-7 somite stage and each frame = 5 minutes. Video shows a single Z plane of a 3D confocal time series.

Supp. video 7: Neural fold fusion in the forebrain region of a *vangl2* morphant zebrafish embryo labeled with membrane-GFP. Movie begins at the 6-7 somite stage and each frame = 5 minutes. Video shows a single Z plane of a 3D confocal time series.

Supp. video 8: Optical transverse section through the posterior forebrain region of a wild-type zebrafish embryo labeled with membrane-GFP. Movie begins at the 4-somite stage and each frame = 5 minutes. Video shows a single Z plane of a 3D confocal time series.

Supp. video 9: Optical transverse section through the posterior forebrain region of a *vangl2* morphant zebrafish embryo labeled with membrane-GFP. Movie begins at the 4-somite stage and each frame = 5 minutes. Video shows a single Z plane of a 3D confocal time series.
